# High-order methods beyond the classical complexity bounds: inexact high-order proximal-point methods

**DOI:** 10.1007/s10107-023-02041-4

**Published:** 2024-01-04

**Authors:** Masoud Ahookhosh, Yurii Nesterov

**Affiliations:** 1 Department of Mathematics, University of Antwerp, Middelheimlaan 1, 2020 Antwerp, Belgium; 2 Center for Operations Research and Econometrics (CORE) and Department of Mathematical Engineering (INMA), Catholic University of Louvain (UCL), 34voie du Roman Pays, 1348 Louvain-la-Neuve, Belgium

**Keywords:** Convex composite optimization, High-order proximal-point operator, Bi-level optimization framework, Lower complexity bounds, Optimal methods, Superfast methods, 90C25, 90C06, 49J52, 65K05, 65Y20

## Abstract

We introduce a *Bi-level OPTimization* (BiOPT) framework for minimizing the sum of two convex functions, where one of them is smooth enough. The BiOPT framework offers three levels of freedom: (i) choosing the order *p* of the proximal term; (ii) designing an inexact *p*th-order proximal-point method in the upper level; (iii) solving the auxiliary problem with a lower-level non-Euclidean method in the lower level. We here regularize the objective by a $(p+1)$th-order proximal term (for arbitrary integer $p\ge 1$) and then develop the generic inexact high-order proximal-point scheme and its acceleration using the standard estimating sequence technique at the upper level. This follows at the lower level with solving the corresponding *p*th-order proximal auxiliary problem inexactly either by one iteration of the *p*th-order tensor method or by a lower-order non-Euclidean composite gradient scheme. Ultimately, it is shown that applying the accelerated inexact *p*th-order proximal-point method at the upper level and handling the auxiliary problem by the non-Euclidean composite gradient scheme lead to a 2*q*-order method with the convergence rate ${\mathcal {O}}(k^{-(p+1)})$ (for $q=\lfloor p/2\rfloor$ and the iteration counter *k*), which can result to a superfast method for some specific class of problems.

## Introduction

*Motivation* Central to the entire discipline of convex optimization is the concept of complexity analysis for evaluating the efficiency of a wide spectrum of algorithms dealing with such problems; see [21, 26]. For example, under the Lipschitz continuity of the gradient of the objective function, the fastest convergence rate for first-order methods is of ${\mathcal {O}}(k^{-2})$ for the iteration counter *k*; cf. [22, 24]. Likewise, if the objective is twice differentiable with Lipschitz continuous Hessian, the best complexity for second-order methods is of ${\mathcal {O}}(k^{-7/2})$; see [7]. In the recent years, there is an increasing interest to applying high-order methods for both convex and nonconvex problems; see, e.g., [1, 7, 10, 12, 17]. If the objective is *p*-times differentiable with Lipschitz continuous *p*th derivatives, then the fastest convergence rate for *p*th-order methods is of ${\mathcal {O}}(k^{-(3p+1)/2})$; cf. [7].

In general, for convex problems, the classical setting involves a one-to-one correspondence between the methods and problem classes. In other words, there exists and unimprovable complexity bound for a class of methods applied to a class of problems under specific assumptions. In fact, under the Lipschitz continuity of the *p*th derivatives, the *p*th-order methods is called *optimal* if it attains the convergence rate ${\mathcal {O}}(k^{-(3p+1)/2})$, and if a method attains a faster convergence rate (under stronger assumptions than the optimal methods), we call it *superfast*. For example, first-order methods with the convergence rate ${\mathcal {O}}(k^{-2})$ and second-order methods with the convergence rate ${\mathcal {O}}(k^{-7/2})$ are optimal under the Lipschitz continuity of the first and the second derivatives, respectively. Recently, in [30], a *superfast second-order method* with the convergence rate ${\mathcal {O}}(k^{-4})$ has been presented, which is faster than the classical lower bound ${\mathcal {O}}(k^{-7/2})$. The latter method consists of an implementation of a third-order tensor method where its auxiliary problem is handled by a Bregman gradient method requiring second-order oracles, i.e., this scheme is implemented as a second-order method. We note that this method assumes the Lipschitz continuity of third derivatives while the classical second-order methods apply to problems with Lipschitz continuous Hessian. This clearly explains that the convergence rate ${\mathcal {O}}(k^{-4})$ for this method is not a contradiction with classical complexity theory for second-order methods.

One of the classical methods for solving optimization problems is the *proximal-point* method that is given by

1.1 $$\begin{aligned} x_{k+1} = \mathop {\mathrm {arg\,min}}\limits _{x\in {\mathbb {E}}} \left\{ {}h(x)+\tfrac{1}{2\lambda }\Vert x-x_k\Vert ^2{}\right\} , \end{aligned}$$

for the function $h(\cdot )$, a given point $x_k$, and $\lambda >0$. The first appearance of this algorithm dated back to 1970 in the works of Martinet [19, 20], which is further studied by Rockafellar [32] when $\lambda$ is replaced by a sequence of positive numbers $\{\lambda _k\}_{k\ge 0}$. Since its first presentation, this algorithm has been subject of great interest in both Euclidean and non-Euclidean settings, and many extensions has been proposed; for example see [5, 9, 11, 15, 16, 33].

Recently, Nesterov in [29] proposed a *bi-level unconstrained minimization* (BLUM) framework by defining a novel high-order proximal-point operator using a *p*th-order regularization term

$$\begin{aligned} {\textrm{prox}}_{h/H}^p({\bar{x}})=\mathop {\mathrm {arg\,min}}\limits _{x\in {\mathbb {E}}} \left\{ {}h(x)+\tfrac{H}{p+1} \Vert x-{\bar{x}}\Vert ^{p+1}{}\right\} , \end{aligned}$$

see Sect. 2 for more details. This framework consists of two levels, where the upper level involves a scheme using the high-order proximal-point operator, and the lower-level is a scheme for solving the corresponding proximal-point minimization inexactly. Therefore, one has a freedom of choosing the order *p* of the proximal-point operator and can also choose a proper method to approximate the solution of the proximal-point auxiliary problem. Applying this framework to twice smooth unconstrained problems with $p=3$, using an accelerated third-order method at the upper level, and solving the auxiliary problem by a Bregman gradient method lead to a second-order method with the convergence rate ${\mathcal {O}}(k^{-4})$. The main goals of this paper are to extend the results of [29] onto the composite case (i.e., for nonsmooth and constrained problems) and to provide a non-Euclidean method for solving the auxiliary *p*th-order proximal-point problem with an arbitrary $p\ge 1$.

### Content

In this paper, we introduce a *Bi-level OPTimization* (BiOPT) framework that is an extension of the BLUM framework (see [29]) for the convex composite minimization. In our setting, the objective function is the sum of two convex functions, where one of them is smooth enough. At the first step, we regularize the objective function by a power of the Euclidean norm $\Vert \cdot \Vert ^{p+1}$ with $p\ge 1$, following the same vein as (1.1). The resulted mapping is called *high-order proximal-point operator*, which is assumed to be minimized approximately at a reasonable cost. If the first function in our composite objective is smooth enough, in Sect. 2, we show that this auxiliary problem can be inexactly solved by one step of the *p*th-order tensor method (see Sect. 2.1). Afterwards, we show that the basic proximal-point method attains the convergence rate ${\mathcal {O}}(k^{-p})$ (see Sect. 2.2), while its accelerated counterpart obtains the convergence rate ${\mathcal {O}}(k^{-(p+1)})$ (see Sect. 2.3).

We next present our bi-level optimization framework in Sect. 3, which opens up entirely new ground for developing highly efficient algorithms for simple constrained and composite minimization problems. In the upper level, we can choose the order *p* of the proximal-point operator and apply both basic and accelerated proximal-point schemes using the estimation sequence technique. We then assume that the differentiable part of the proximal-point objective is smooth relative to some scaling function (see [9, 18]) and then design a non-Euclidean composite gradient algorithm using a Bregman distance to solve this auxiliary problem inexactly. It is shown that the latter algorithm will be stopped after ${\mathcal {O}}(\log \tfrac{1}{\varepsilon })$ of iterations (for the accuracy parameter $\varepsilon >0$) if the underlying cost function is relatively strongly convex. Hence, choosing a lower-order scaling function for the Bregman distance, there is a possibility to apply lower-order schemes for solving the auxiliary problem that will lead to lower-order methods in our convex composite setting.

Following our BiOPT framework, we finally pick a constant *p* for the *p*th-order proximal-point operator and apply the accelerated method to the composite problem at the upper level. Then, we introduce a high-order scaling function and show that the differentiable part of the proximal-point objective is *L*-smooth relative to this scaling function, for $L>0$. We consequently apply the non-Euclidean composite gradient method to the auxiliary problem, which only needs the *p*th-order oracle for even *p* and the $(p-1)$th-order oracle for odd *p*. Therefore, we end up with a high-order method with the convergence rate of ${\mathcal {O}}(k^{-(p+1)})$ under some suitable assumptions. We emphasize while this convergence rate is faster than the classical lower bound ${\mathcal {O}}(k^{-(3p-2)/2})$ for $p=3$, it is sub-optimal for other choices of *p*. However, we show that our method can overpass the classical optimal rates for some class of structured problems. We finally deliver some conclusion in Sect. 4.

### Notation and generalities

In what follows, we denote by ${\mathbb {E}}$ a finite-dimensional real vector space and by ${\mathbb {E}}^*$ its dual spaced composed by linear functions on ${\mathbb {E}}$. For such a function $s\in {\mathbb {E}}^*$, we denote by $\left\langle {}s{},{}x{}\right\rangle$ its value at $x\in {\mathbb {E}}$.

Let us measure distances in ${\mathbb {E}}$ and ${\mathbb {E}}^*$ in a Euclidean norm. For that, using a self-adjoint positive-definite operator $B:{\mathbb {E}}\rightarrow {\mathbb {E}}^*$ (notation $B=B^* \succ 0$), we define

$$\begin{aligned} \Vert x\Vert =\left\langle {}Bx{},{}x{}\right\rangle ^{1/2},\quad x\in {\mathbb {E}},\quad \Vert g\Vert _{*} = \left\langle {}g{},{}B^{-1}g{}\right\rangle ^{1/2}, \quad g\in {\mathbb {E}}^*. \end{aligned}$$

Sometimes, it will be convenient to treat $x\in {\mathbb {E}}$ as a linear operator from ${\mathbb {R}}$ to ${\mathbb {E}}$, and $x^*$ as a linear operator from ${\mathbb {E}}^*$ to ${\mathbb {R}}$. In this case, $xx^*$ is a linear operator from ${\mathbb {E}}^*$ to ${\mathbb {E}}$, acting as follows:

$$\begin{aligned} (xx^*)g = \left\langle {}g{},{}x{}\right\rangle x\in {\mathbb {E}},\quad g\in {\mathbb {E}}^*. \end{aligned}$$

For a smooth function $f:{\mathbb {E}}\rightarrow {\mathbb {R}}$ denote by $\nabla f(x)$ its gradient, and by $\nabla ^2 f(x)$ its Hessian evaluated at the point $x\in {\mathbb {E}}$. Note that

$$\begin{aligned} \nabla f(x)\in {\mathbb {E}}^*, \quad \nabla ^2 f(x)h\in {\mathbb {E}}^*, \quad x,h\in {\mathbb {E}}. \end{aligned}$$

We denote by $\ell _{{\bar{x}}}(\cdot )$ the linear model of convex function $f(\cdot )$ at point ${\bar{x}}\in {\mathbb {E}}$ given by

1.2 $$\begin{aligned} \ell _{{\bar{x}}}(x) = f({\bar{x}})+\left\langle {}\nabla f({\bar{x}}){},{}x-{\bar{x}}{}\right\rangle , \quad x\in {\mathbb {E}}. \end{aligned}$$

Using the above norm, we can define the standard Euclidean prox-functions

$$\begin{aligned} d_{p+1}(x)=\tfrac{1}{p+1}\Vert x\Vert ^{p+1}, \quad x\in {\mathbb {E}}. \end{aligned}$$

where $p\ge 1$ is an integer parameter. These functions have the following derivatives:

1.3 $$\begin{aligned} \nabla d_{p+1}(x)&= \Vert x\Vert ^{p-1}Bx, \quad x\in {\mathbb {E}}, \nonumber \\ \nabla ^2 d_{p+1}(x)&= \Vert x\Vert ^{p-1}B+(p-1)\Vert x\Vert ^{p-3}Bxx^*B \succeq \Vert x\Vert ^{p-1}B. \end{aligned}$$

Note that function $d_{p+1}(\cdot )$ is uniformly convex (see, for example, [26, Lemma 4.2.3]):

1.4 $$\begin{aligned} d_{p+1}(y) \ge d_{p+1}(x)+\left\langle {}\nabla d_{p+1}(x){},{}y-x{}\right\rangle +\tfrac{1}{p+1} \left( \tfrac{1}{2}\right) ^{p-1} \Vert y-x\Vert ^{p+1}, \quad x,y\in {\mathbb {E}}. \end{aligned}$$

In what follows, we often work with directional derivatives. For $p\ge 1$, denote by

$$\begin{aligned} D^pf(x)[h_1,\ldots ,h_p] \end{aligned}$$

the directional derivative of function *f* at *x* along directions $h_i\in {\mathbb {E}}$, $i= 1,\ldots ,p$. Note that $D^pf(x)[\cdot ]$ is a symmetric *p*-linear form. Its norm is defined in a standard way:

1.5 $$\begin{aligned} \Vert D^pf(x)\Vert = \max _{h_1,\ldots ,h_p} \left\{ {}\left| D^pf(x)[h_1,\ldots ,h_p]\right| : \Vert h_i\Vert \le 1,\; i=1,\ldots ,p{}\right\} . \end{aligned}$$

If all directions $h_1,\ldots ,h_p$ are the same, we apply the notation

$$\begin{aligned} D^pf(x)[h]^p,\quad h\in {\mathbb {E}}. \end{aligned}$$

Note that, in general, we have (see, for example, [31, Appendix 1])

1.6 $$\begin{aligned} \Vert D^pf(x)\Vert = \max _{h} \left\{ {}\left| D^pf(x)[h]^p\right| : \Vert h\Vert \le 1{}\right\} . \end{aligned}$$

In this paper, we work with functions from the problem classes ${\mathcal {F}}_p$, which are convex and *p* times continuously differentiable on ${\mathbb {E}}$. Denote by $M_p(f)$ its uniform upper bound for its *p*th derivative:

1.7 $$\begin{aligned} M_p(f)=\sup _{x\in {\mathbb {E}}} \Vert D^pf(x)\Vert . \end{aligned}$$

## Inexact high-order proximal-point methods

Let function $f:{\mathbb {E}}\rightarrow {\mathbb {R}}$ be closed convex and smooth enough and let $\psi :{\mathbb {E}}\rightarrow {\mathbb {R}}$ be a simple closed convex function such that ${\textrm{dom}}\psi \subseteq {\textrm{int}}({\textrm{dom}}f)$. We now consider the convex composite minimization problem

2.1 $$\begin{aligned} \min _{x\in {\textrm{dom}}\psi }~\left\{ {}F(x)=f(x)+\psi (x){}\right\} , \end{aligned}$$

where it is assumed that (2.1) has at least one optimal solution $x^*\in {\textrm{dom}}\psi$ and $F^*=F(x^*)$. This class of problems is general enough to encompass many practical problems from many application fields such as signal and image processing, machine learning, statistics, and so on. In particular, for the simple closed convex set $Q\subseteq {\mathbb {E}}$, the simple constrained problem

2.2 $$\begin{aligned} \begin{array}{ll} \min &{}\quad f(x) \\ \mathrm {s.t.} &{}\quad x\in Q \end{array} \end{aligned}$$

can be rewritten in the form (2.1), i.e.,

2.3 $$\begin{aligned} \min _{x\in {\textrm{dom}}\psi }\; f(x)+\delta _Q(x), \end{aligned}$$

where $\delta _Q(\cdot )$ is the indicator function of the set *Q* given by

$$\begin{aligned} \delta _Q(x)=\left\{ \begin{array}{ll} 0 &{}\quad \textrm{if}\;x\in Q, \\ +\infty &{}\quad \textrm{if}\;x\not \in Q. \end{array} \right. \end{aligned}$$

Let us define the *pth-order composite proximal-point operator*

2.4 $$\begin{aligned} {\textrm{prox}}_{F/H}^p({\bar{x}})=\mathop {\mathrm {arg\,min}}\limits _{x\in {\textrm{dom}}\psi } \left\{ {}f(x)+\psi (x)+H d_{p+1}(x-{\bar{x}}){}\right\} , \end{aligned}$$

for $H>0$ and $p\ge 1$, which is an extension of the *p*th-order proximal-point operator given in [29]. Moreover, if $p=1$, it reduces to the classical proximal operator

$$\begin{aligned} \textrm{prox}_{F/H}({\bar{x}}) = \mathop {\mathrm {arg\,min}}\limits _{x\in {\textrm{dom}}\psi } \left\{ {}f(x)+\psi (x)+\tfrac{H}{2} \Vert x-{\bar{x}}\Vert ^2{}\right\} . \end{aligned}$$

Our main objective is to investigate the global rate of convergence of high-order proximal-point methods in accelerated and non-accelerated forms, where we approximate the proximal-point operator (2.4) and study the complexity of such approximation. To this end, let us introduce the set of *acceptable solutions* of (2.4) by

2.5 $$\begin{aligned} {\mathcal {A}}_p^H({\bar{x}},\beta )&= \big \{(x,g)\in {\textrm{dom}}\psi \times {\mathbb {E}}^* : g\in \partial \psi (x), \; \Vert \nabla f_{{\bar{x}},p}^H(x)+g\Vert _* \nonumber \\&\le \beta \Vert \nabla f(x)+g\Vert _*\big \}, \end{aligned}$$

where

2.6 $$\begin{aligned} f_{{\bar{x}},p}^H(x)=f(x)+H d_{p+1}(x-{\bar{x}}), \end{aligned}$$

where $\beta \in [0,1)$ is the tolerance parameter. Note that if $\psi \equiv 0$, then the set ${\mathcal {A}}_p^H({\bar{x}},\beta )$ leads to *inexact acceptable solutions* for the problem (2.4), which was recently studied for smooth convex problems in [29]. Let us emphasize that extending the definition of inexact acceptable solutions from [29] for nonsmooth functions is not a trivial task because not all subgradients $g\in \partial \psi (x)$ satisfy the inequality (2.5). In the more general setting of the composite minimization, we address this issue in Sect. 3.1 using a non-Euclidean composite gradient scheme that suggests which subgradient $g\in \partial \psi (x)\ne \emptyset$ can be explicitly used in (2.5).

Since function $F(\cdot )$ is convex and $d_{p+1}(\cdot )$ is uniformly convex, the minimization problem (2.4) has a unique solution that we assume to be computable at reasonable cost. Let us first see how the exact solution of (2.4) satisfies (2.5). The first-order optimality conditions for (2.4) ensure that

$$\begin{aligned} H \Vert T-{\bar{x}}\Vert ^{p-1}B({\bar{x}}-T)- \nabla f(T) \in \partial \psi (T). \end{aligned}$$

Thus, for $g=H \Vert T-{\bar{x}}\Vert ^{p-1}B({\bar{x}}-T)- \nabla f(T)$, the inequality in (2.5) holds with any $\beta \in [0,1)$, i.e., $({\textrm{prox}}_{F/H}^p({\bar{x}}),g)\in {\mathcal {A}}_p^H({\bar{x}},\beta )$. Furthermore, since $\nabla f_{{\bar{x}},p}^H({\bar{x}})=\nabla f({\bar{x}})$, we have $(\bar{x},g)\not \in {\mathcal {A}}_p^H({\bar{x}},\beta )$ except if ${\bar{x}} = x^*$. In the next subsection, we show that an acceptable approximation of the operator (2.4) can be computed by applying one step of the *p*th-order tensor method (see [27]) satisfying (2.5), while a lower-order method will be presented in Sect. 3.1. Let us highlight here that we are not able to find an inexact solution in the sense of (2.5) for all points ${\bar{x}}$ in a neighbourhood of the solution $x^*$; however its exact solution always satisfies this inequality. We study this in the following example.

### Example 2.1

Let us consider the minimization of function $f:{\mathbb {R}}\rightarrow {\mathbb {R}}$ given by $f(x)=x$ over the set $Q=\left\{ {}x\in {\mathbb {R}}:x\ge 0{}\right\}$, where $x^*=0$ is its unique solution. The indicator function of the set *Q* is given by $\psi :{\mathbb {R}}\rightarrow {\mathbb {R}}$ that is

$$\begin{aligned} \psi (x) = \delta _Q(x) = \left\{ \begin{array}{ll} 0 &{} \quad \textrm{if}\; x\ge 0, \\ +\infty &{}\quad \textrm{if}\; x<0, \end{array} \right. \end{aligned}$$

where its subdifferential is given by

$$\begin{aligned} \partial \psi (x) = \left\{ \begin{array}{ll} (-\infty ,0] &{} \quad \textrm{if}\; x=0, \\ \{0\} &{} \quad \textrm{if}\; x>0,\\ \emptyset &{} \quad \textrm{if}\; x<0. \end{array} \right. \end{aligned}$$

Let us set $H=1, B=1, p=3, {\bar{x}} \ne 0$. Hence, $f_{{\bar{x}}, H}^3(x)=x+\tfrac{1}{4} |x-{\bar{x}}|^4$ that for $x\ge 0$ and $g\in \partial \psi (x)$ yield $\Vert \nabla f_{{\bar{x}}, H}^3(x)+g\Vert _*=|1+g+(x-{\bar{x}})^3|,\;\Vert \nabla f(x)+g\Vert _*=|1+g|$. Therefore, for $\beta \in [0,1)$, the inequality $\Vert \nabla f_{{\bar{x}}, H}^3(T)+g\Vert _*\le \beta \Vert \nabla f(T)+g\Vert _*$ leads to $|1+g+(T-\bar{x})^3|\le \beta |1+g|$, i.e.,

$$\begin{aligned} {\bar{x}}-\root 3 \of {1+g+\beta |1+g|} \le T \le \bar{x}-\root 3 \of {1+g-\beta |1+g|},\quad T\ge 0. \end{aligned}$$

**Fig. 1:**
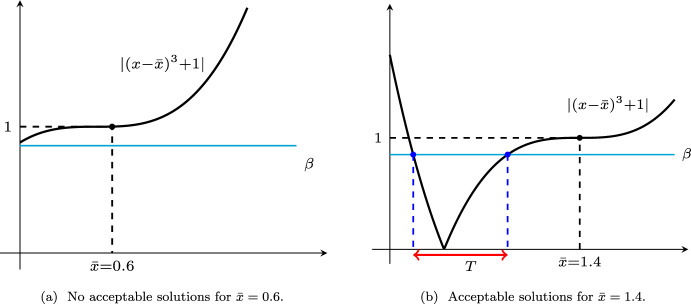
Subfigure (**a**) shows that for ${\bar{x}} <\root 3 \of {1-\beta }$, only the exact solution of the auxiliary problem satisfies (2.5), and Subfigure (**b**) illustrates the set of solutions for ${\bar{x}}=1.4$ and $\beta =0.85$ satisfying $\bar{x}\ge \root 3 \of {1+\beta }$

It is clear that there is no $T>0$ (i.e., $g=0$) such that the right-hand side inequality holds if we have ${\bar{x}} <\root 3 \of {1-\beta }$ [see Subfigure (a) of Fig. 1]. In this case, only the exact solution $T=0$ of the auxiliary problem satisfies the inequality (2.5). Indeed, $(T,g)\in {\mathcal {A}}_p^H(\bar{x},\beta )$ if we have

$$\begin{aligned} T \in \left\{ \begin{array}{ll} \left[ {\bar{x}}-\root 3 \of {1+g+\beta |1+g|},\bar{x}-\root 3 \of {1+g-\beta |1+g|}\right] &{} \quad \textrm{if}\; \bar{x}-\root 3 \of {1+g+\beta |1+g|}\ge 0, \\ \left[ 0,{\bar{x}}-\root 3 \of {1+g-\beta |1+g|}\right] &{} \quad \textrm{if}\; {\bar{x}}-\root 3 \of {1+g+\beta |1+g|}<0, \end{array} \right. \end{aligned}$$

which we illustrate in Subfigure (b) of Fig. 1 for some special choices of $\beta$ and ${\bar{x}}$.

We first present the following lemma, which is a direct consequence of the definition of acceptable solutions (2.5).

### Lemma 2.2

(Properties of acceptable solutions) Let $(T,g)\in {\mathcal {A}}_p^H({\bar{x}},\beta )$ for some $g\in \partial \psi (T)$. Then, we have

2.7 $$\begin{aligned}{} & {} (1-\beta )\Vert \nabla f(T)+g\Vert _* \le H \Vert T-{\bar{x}}\Vert ^p \le (1+\beta )\Vert \nabla f(T)+g\Vert _*, \end{aligned}$$

2.8 $$\begin{aligned}{} & {} \left\langle {}\nabla f(T)+g{},{}{\bar{x}}-T{}\right\rangle \ge \tfrac{H}{1+\beta } \Vert T-\bar{x}\Vert ^{p+1}. \end{aligned}$$

If additionally $\beta \le \tfrac{1}{p}$, then

2.9 $$\begin{aligned} \left\langle {}\nabla f(T)+g{},{}{\bar{x}}-T{}\right\rangle \ge \left( \tfrac{1-\beta }{H}\right) ^{1/p} \Vert \nabla f(T)+g\Vert _*^{\tfrac{p+1}{p}}. \end{aligned}$$

### Proof

From (2.5) and the reverse triangle inequality, we obtain

$$\begin{aligned} \big |H\Vert T-{\bar{x}}\Vert ^p-\Vert \nabla f(T)+g\Vert _*\big |&\le \Vert \nabla f(T)+H\Vert T-{\bar{x}}\Vert ^{p-1}B(T-{\bar{x}})\\&\quad +g\Vert _* \le \beta \Vert \nabla f(T)+g\Vert _*, \end{aligned}$$

i.e., the inequality (2.7) holds. Squaring both sides of the inequality in (2.5), we come to

$$\begin{aligned}&\Vert \nabla f(T)+g\Vert _*^2+2H\Vert T-{\bar{x}}\Vert ^{p-1} \left\langle {}\nabla f(T)+g{},{}B(T-{\bar{x}}){}\right\rangle \\&\quad +H^2\Vert T-{\bar{x}}\Vert ^{2p} \le \beta ^2 \Vert \nabla f(x)+g\Vert _*^2, \end{aligned}$$

leading to

2.10 $$\begin{aligned} \left\langle {}\nabla f(T)+g{},{}B({\bar{x}}-T){}\right\rangle&\ge \tfrac{1-\beta ^2}{2H\Vert T-{\bar{x}}\Vert ^{p-1}} \Vert \nabla f(T)+g\Vert _*^2+\tfrac{H}{2}\Vert T-{\bar{x}}\Vert ^{p+1} \nonumber \\&\ge \tfrac{H(1-\beta ^2)}{2(1+\beta )^2} \Vert T-\bar{x}\Vert ^{p+1}+\tfrac{H}{2}\Vert T-{\bar{x}}\Vert ^{p+1} \nonumber \\&= \tfrac{H}{(1+\beta )} \Vert T-{\bar{x}}\Vert ^{p+1}, \end{aligned}$$

giving (2.8). Let us consider the function $\zeta :{\mathbb {R}}_+\rightarrow {\mathbb {R}}$ with $\zeta (r)=\tfrac{1-\beta ^2}{2H r^{p-1}} \Vert \nabla f(T)+g\Vert _*^2+\tfrac{H}{2} r^{p+1}$, which is the right-hand side of the inequality (2.10) with $r=\Vert T-{\bar{x}}\Vert$. From the inequality (2.7), we obtain $r\ge \widehat{r}= \left( \tfrac{1-\beta }{H}\Vert \nabla f(T)+g\Vert _*\right) ^{1/p}$. Taking the derivative of $\zeta$ at $\widehat{r}$ and $\beta \le \tfrac{1}{p}$, we get

$$\begin{aligned} \zeta '(\widehat{r})&= \left( \tfrac{(1-p)(1+\beta )}{2}+\tfrac{(p+1)(1-\beta )}{2}\right) \Vert \nabla f(T)+g\Vert _* \\&= (1-\beta p)\Vert \nabla f(T)+g\Vert _*\ge 0. \end{aligned}$$

Together with (2.10), this implies (2.9). $\square$

### Solving (2.4) with *p*th-order tensor methods

In this section, we assume that $f(\cdot )$ is *p*th-order differentiabe with $M_{p+1}(f)<+\infty$ and show that an acceptable solution satisfying the inequality (2.5) can be obtained by applying one step of the tensor method given in [27].

The Taylor expansion of the function $f(\cdot )$ at $x\in {\mathbb {E}}$ is denoted by

$$\begin{aligned} \varOmega _{x,p}(y) = f(x)+\sum _{k=1}^p\tfrac{1}{k!} D^k f(x)[y-x]^k,\quad y\in {\mathbb {E}}, \end{aligned}$$

and it holds that

2.11 $$\begin{aligned} \Vert \nabla f(y)-\nabla \varOmega _{x,p}(y)\Vert _* \le \tfrac{M_{p+1}(f)}{p!} \Vert y-x\Vert ^p. \end{aligned}$$

For $M>0$, let us define the *augmented Taylor approximation* as

$$\begin{aligned} \widehat{\varOmega }_{x,p}(y) = \varOmega _{x,p}(y)+\tfrac{M}{(p+1)!} \Vert y-x\Vert ^{p+1}. \end{aligned}$$

Note that if $M\ge M_{p+1}(f)$, then $F(y)\le {\widehat{\varOmega }}_{x,p}(y)+\psi (y)$, which is a uniform upper bound for $F(\cdot )$. In the case $M\ge p M_{p+1}(f)$, the function ${\widehat{\varOmega }}_{x,p}(y)+\psi (y)$ is convex, as confirmed by [27, Theorem 1], which implies that one will be able to minimize the problem (2.1) by the *tensor step*, i.e.,

2.12 $$\begin{aligned} T_{p,M}^{f,g}(x)=\displaystyle \mathop {\mathrm {arg\,min}}\limits _{y\in {\textrm{dom}}\psi } {\widehat{\varOmega }}_{x,p}(y)+\psi (y). \end{aligned}$$

We next show that an approximate solution of (2.12) can be employed as an acceptable solution of the proximal-point operator (2.4) by the inexact *p*th-order tensor method proposed in [14, 27].

#### Lemma 2.3

(Acceptable solutions by the tensor method (2.12)) Let $(1-\gamma )M>M_{p+1}(f)$ and the approximate solution *T* of (2.12) satisfies

2.13 $$\begin{aligned} \Vert \nabla {\widehat{\varOmega }}_{x,p}(T)+ g\Vert _* \le \tfrac{\gamma }{1+\gamma }\Vert \nabla \varOmega _{x,p}(T)+ g\Vert _*, \end{aligned}$$

for some $g\in \partial \psi (T)$ and $\gamma \in \left[ 0, \tfrac{\beta }{1+\beta }\right)$. Then, for point $T=T_{p,M}^{f,g}(x)$, it holds

2.14 $$\begin{aligned} \Vert \nabla f(T)+\tfrac{M}{p!}\nabla d_{p+1}(T-x)+g\Vert _* \le \tfrac{M_{p+1}(f)+\gamma M}{(1-\gamma )M-M_{p+1}(f)} \Vert \nabla f(T)+ g\Vert _*. \end{aligned}$$

#### Proof

It follows from (2.13) that

$$\begin{aligned} \tfrac{\gamma }{1+\gamma }\Vert \nabla \varOmega _{x,p}(T)+ g\Vert _* \ge \Vert \nabla {\widehat{\varOmega }}_{x,p}(T)+ g\Vert _* \ge \Vert \nabla \varOmega _{x,p}(T)+ g\Vert _*-\tfrac{M}{p!}\Vert T-x\Vert ^p, \end{aligned}$$

which consequently implies

$$\begin{aligned} \Vert \nabla \varOmega _{x,p}(T)+ g\Vert _* \le (1+\gamma ) \tfrac{M}{p!}\Vert T-x\Vert ^p, \end{aligned}$$

for some $g\in \partial \psi (T)$. Together with (1.3), (2.11), and (2.13), this yields

$$\begin{aligned} \tfrac{M_{p+1}(f)}{p!} \Vert T-x\Vert ^p&\ge \Vert \nabla f(T)-\nabla \varOmega _{x,p}(T)\Vert _* \\&= \Vert \nabla f(T)-\nabla {\widehat{\varOmega }}_{x,p}(T)+\tfrac{M}{p!}\nabla d_{p+1}(T-x)\Vert _*\\&= \Vert \nabla f(T)+\tfrac{M}{p!}\nabla d_{p+1}(T-x)+g-(\nabla {\widehat{\varOmega }}_{x,p}(T)+g)\Vert _*\\&\ge \Vert \nabla f(T)+\tfrac{M}{p!}\nabla d_{p+1}(T-x)+g\Vert _*-\Vert \nabla {\widehat{\varOmega }}_{x,p}(T)+g\Vert _*\\&\ge \Vert \nabla f(T)+\tfrac{M}{p!}\nabla d_{p+1}(T-x)+g\Vert _*-\tfrac{\gamma M}{p!}\Vert T-x\Vert ^p\\&\ge \Vert \tfrac{M}{p!}\nabla d_{p+1}(T-x)\Vert _*-\Vert \nabla f(T)+g\Vert _*-\tfrac{\gamma M}{p!}\Vert T-x\Vert ^p, \end{aligned}$$

implying $\tfrac{1}{p!} \Vert T-x\Vert ^p\le \tfrac{1}{(1-\gamma ) M-M_{p+1}(f)} \Vert \nabla f(T)+g\Vert _*$. This and the inequality

$$\begin{aligned} \Vert \nabla f(T)+\tfrac{M}{p!}\nabla d_{p+1}(T-x)+g\Vert _*&\le \tfrac{M_{p+1}(f)+\gamma M}{p!} \Vert T-x\Vert ^p, \end{aligned}$$

obtained in the above chain lead to the desired result (2.14). $\square$

We note that setting $M=\tfrac{1+\beta }{\beta (1-\gamma )-\gamma }M_{p+1}(f)$ and $H=\tfrac{M}{p!}$, the inequality (2.14) can be rewritten in the form

$$\begin{aligned} \Vert \nabla f(T)+H\nabla d_{p+1}(T-x)+g\Vert _* \le \beta \Vert \nabla f(T)+g\Vert _*, \end{aligned}$$

which implies $(T,g)\in {\mathcal {A}}_p^H(x,\beta )$. In order to illustrate the results of Lemma 2.3, we study the following one-dimensional example.

#### Example 2.4

Let us consider the minimization of the one-dimensional function $F:{\mathbb {R}}\rightarrow {\mathbb {R}}$ given by $F(x)=x^4+|x|$, where $x^*=0$ is its unique solution. In the setting of the problem (2.1), we have $f(x)=x^4$ and $\psi (x)=|x|$. Let us set $p=3$, i.e., we have $M_4(f)=24$ and

$$\begin{aligned} \varOmega _{x,3}(y)&= x^4+4x^3(y-x)+6x^2(y-x)^2+4x(y-x)^3,\\ \widehat{\varOmega }_{x,3,M}(y)&=\varOmega _{x,3}(y)+\tfrac{M}{24} (y-x)^4, \end{aligned}$$

where $$M=1.9 M_4(f)$$. Thus,

$$\begin{aligned} \varOmega _{x,3}'(y)= 4x^3+12x^2(y-x)+12x(y-x)^2,\quad \widehat{\varOmega }_{x,3,M}'(y)=\varOmega _{x,3}'(y)+\tfrac{M}{6} (y-x)^3. \end{aligned}$$

**Fig. 2:**
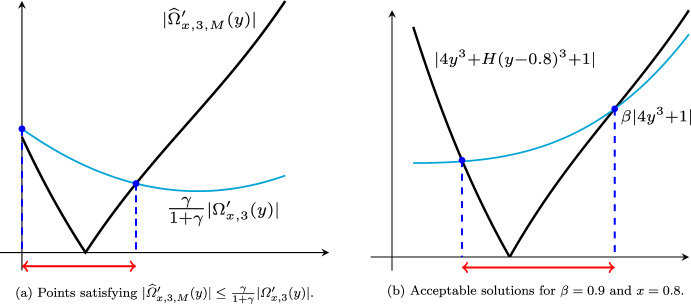
Subfigure (**a**) stands for the set of points *y* satisfying the inequality $|\widehat{\varOmega }'_{x,3,M}(y)|\le \tfrac{\gamma }{1+\gamma }|\varOmega '_{x,3}(y)|$ with $x=0.8$, $\gamma =8/19$, and $\beta =0.9$, and Subfigure (**b**) illustrates the set of acceptable solutions for $x=0.8$ and $\beta =0.9$

Setting $\gamma =\tfrac{8}{19}\in [0,\tfrac{9}{19})$ and $x=0.8$, we illustrate the feasible area of $|\widehat{\varOmega }'_{x,3,M}(y)|\le \tfrac{\gamma }{1+\gamma }|\varOmega '_{x,3}(y)|$ and acceptable solutions in Subfigures (a) and (b) of Fig. 2, respectively. We note that with our choice of $\gamma$ and *M*, we have $(1-\gamma )M>M_4(f)$, which implies that all assumptions of Lemma 2.3 are valid.

In Sect. 3, we further extend our discussion concerning the computation of an acceptable solution ${\mathcal {A}}_p^H(\bar{x},\beta )$ for the *p*th-order proximal-point problem (2.4) by the lower-level methods.

### Inexact high-order proximal-point method

In this section, we introduce our inexact high-order proximal-point method for the composite minimization (2.1) and verify its rate of convergence.

We now consider our first inexact high-order proximal-point scheme that generates a sequence of iterations satisfying

2.15 $$\begin{aligned} (T_k,g_{k+1}) \in {\mathcal {A}}_p^H(x_k,\beta ), \end{aligned}$$

for $g_{k+1}\in \partial \psi (T_k)$ which we summarize in Algorithm 1.
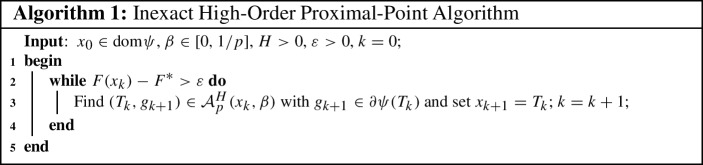


In order to verify the the convergence rate of Algorithm 1, we need the next lemma, which was proved in [28, Lemma 11].

#### Lemma 2.5

[28, Lemma 11] Let $\{\xi _k\}_{k\ge 0}$ be a sequence of positive numbers satisfying

2.16 $$\begin{aligned} \xi _k-\xi _{k+1} \ge \xi _{k+1}^{1+\alpha },\quad k\ge 0, \end{aligned}$$

for $\alpha \in (0,1]$. Then, for $k\ge 0$, the following holds

2.17 $$\begin{aligned} \xi _k \le \tfrac{\xi _0}{\left( 1+\tfrac{\alpha k}{1+\alpha }\log (1+\xi _0^\alpha )\right) ^{1/\alpha }} \le \left( \left( 1+\tfrac{1}{\alpha }\right) (1+\xi _0^\alpha )\cdot \tfrac{1}{k}\right) ^{1/\alpha }. \end{aligned}$$

Let us investigate the rate of convergence of Algorithm 1. Let us first define the *radius of the initial level set* of the function $\psi$ in (2.1) as $D_0= \max _{x\in {\textrm{dom}}\psi } \left\{ {}\Vert x-x^*\Vert :F(x)\le F(x_0){}\right\} <+\infty$.

#### Theorem 2.6

(Convergence rate of Algorithm 1) Let the sequence $\{x_k\}_{k\ge 0}$ be generated by the inexact high-order proximal-point method (2.15) with $\beta \in [0,1/p]$. Then, for $k\ge 0$, we have

2.18 $$\begin{aligned} F(x_k)-F^* \le \tfrac{1}{2} \left( \tfrac{1}{1-\beta } H D_0^{p+1}+F(x_0)-F^*\right) \left( \tfrac{2p+2}{k}\right) ^p. \end{aligned}$$

#### Proof

From the convexity of $\psi (\cdot )$ and (2.9), we obtain

$$\begin{aligned} F(x_k)-F(x_{k+1})&\ge \left\langle {}\nabla f(x_{k+1})+g_{k+1}{},{}x_{k+1}-x_k{}\right\rangle \\&\ge \left( \tfrac{1-\beta }{H}\right) ^{1/p} \Vert \nabla f(x_{k+1})+g_{k+1}\Vert _*^{\tfrac{p+1}{p}}, \end{aligned}$$

with $g_{k+1}\in \partial \psi (x_{k+1})$ and $(x_{k+1},g_{k+1}) \in {\mathcal {A}}_p^H(x_k,\beta )$. By Cauchy–Schwartz inequalitiy, we get

$$\begin{aligned} F(x_{k+1})-F^*&\le \left\langle {}\nabla f(x_{k+1})+g_{k+1}{},{}x_{k+1}-x^*{}\right\rangle \\&\le \Vert \nabla f(x_{k+1})+g_{k+1}\Vert _*\Vert x_{k+1}-x^*\Vert \\&\le D_0 \Vert \nabla f(x_{k+1})+g_{k+1}\Vert _*. \end{aligned}$$

It follow from the last two inequalities, that

$$\begin{aligned} F(x_k)-F(x_{k+1}) \ge \left( \tfrac{1-\beta }{H D_0^{p+1}}\right) ^{1/p} \left( F(x_{k+1})-F^*\right) ^{\tfrac{p+1}{p}}. \end{aligned}$$

Setting $\xi _k=\tfrac{1-\beta }{H D_0^{p+1}}(F(x_k)-F^*)$ and $\alpha =1/p$, we see that the condition (2.16) is satisfied for all $k\ge 0$. Therefore, from Lemma 2.5, we have

$$\begin{aligned} \xi _k \le \left( \left( 1+\tfrac{1}{\alpha }\right) (1+\xi _0^\alpha )\cdot \tfrac{1}{k}\right) ^{\tfrac{1}{\alpha }} \le \left( 1+\tfrac{1}{\alpha }\right) ^{\tfrac{1}{\alpha }} 2^{\tfrac{1-\alpha }{\alpha }}(1+\xi _0) \left( \tfrac{1}{k}\right) ^{\tfrac{1}{\alpha }}, \end{aligned}$$

adjusting (2.18). $$\square $$

### Accelerated inexact high-order proximal-point method

In this section, we accelerate the scheme (2.15) by applying a variant of the *standard estimating sequences technique*, which has been used as a standard tool for accelerating first- and second-order methods; see, e.g., [2, 8, 22–26].

Let $\{A_k\}_{k\ge 0}$ be a sequence of positive numbers generated by $A_{k+1}=A_k+a_{k+1}$ for $a_k>0$. The idea of the estimating sequences techniques is to generate a sequence of estimating functions $\{\varPsi _k(x)\}_{k\ge 0}$ of $F(\cdot )$ in such a way that, at each iteration $k\ge 0$, the inequality

2.19 $$\begin{aligned} A_k F(x_k)\le \varPsi _k^*\equiv \min _{x\in {\textrm{dom}}\psi } \varPsi _k(x), \quad k\ge 0 \end{aligned}$$

holds true. Let us set $c_p=\left( \tfrac{1-\beta }{H}\right) ^{1/p}$. Following [29, 30], we set

2.20 $$\begin{aligned} A_{k}= \left( \tfrac{c_p}{2}\right) ^p \left( \tfrac{k}{p+1}\right) ^{p+1}, \quad a_{k+1}=A_{k+1}-A_k, \quad k\ge 0. \end{aligned}$$

For $x_0, y_k\in {\mathbb {E}}$ and $(T_k,g_{k+1})\in {\mathcal {A}}_p^H(y_k,\beta )$, let us define the *estimating sequence*

2.21 $$\begin{aligned} \varPsi _{k+1}(x)=\left\{ \begin{array}{ll} d_{p+1}(x-x_0) &{}\quad \textrm{if}\;k=0,\\ \varPsi _k(x)+a_{k+1}[\ell _{T_k}(x)+\psi (x)] &{}\quad \textrm{if}\; k\ge 1. \end{array} \right. \end{aligned}$$

#### Lemma 2.7

Let the sequence $\{\varPsi _k(x)\}_{k\ge 0}$ be generated by (2.21) and $\upsilon _k=\mathop {\mathrm {arg\,min}}\limits _{x\in {\mathbb {E}}} \varPsi _k(x)$. Then, it holds that

2.22 $$\begin{aligned} A_k F(x)+ d_{p+1}(x-x_0)&\ge \varPsi _k(x)\nonumber \\&\ge \varPsi _k^*+\tfrac{1}{p+1}\left( \tfrac{1}{2}\right) ^{p-1} \Vert x-\upsilon _k\Vert ^{p+1}, \quad \forall x\in {\textrm{dom}}\psi ,~ k\ge 0. \end{aligned}$$

#### Proof

The proof is given by induction on *k*. For $k=0$, $\varPsi _0=d_{p+1}(x-x_0)$ and so (2.22) holds. We now assume that (2.22) holds for *k* and show it for $k+1$. Then, it follows from (2.21) and the subgradient inequality that

$$\begin{aligned} \varPsi _{k+1}(x)&=\varPsi _k(x)+a_{k+1}[\ell _{x_{k+1}}(x)+\psi (x)]\\&\le A_k F(x)+ d_{p+1}(x-x_0)+a_{k+1}[\ell _{x_{k+1}}(x)+\psi (x)]\\&\le A_k F(x)+ d_{p+1}(x-x_0)+a_{k+1}F(x), \end{aligned}$$

leading to (2.22) for $k+1$. The right-hand side inequality in (2.22) is a direct consequence of the definition of $\varPsi _k(\cdot )$ and (1.4). $\square$

We next present an accelerated version of the scheme (2.5).
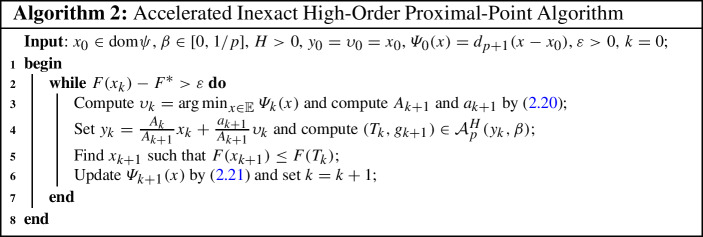


In the subsequent result, we investigate the convergence rate of the sequence generated by the accelerated inexact high-order proximal-point method (Algorithm 2).

#### Theorem 2.8

(Convergence rate of Algorithm 2) Let the sequence $\{x_k\}_{k\ge 0}$ be generated by Algorithm 2 with $\beta \in [0,1/p]$. Then, the following statements hold:

(i) for all $k\ge 0$, the inequality (2.19) holds;
(ii) for all $k\ge 0$,
  2.23 $$\begin{aligned} F(x_k)-F^* \le \tfrac{H}{2(1-\beta )} d_{p+1}(x_0-x^*) \left( \tfrac{2p+2}{k}\right) ^{p+1}; \end{aligned}$$
(iii) $d_{p+1}(\upsilon _k-x^*) \le 2^{p-1} d_{p+1}(x_0-x^*)$, for all $k\ge 0$.

#### Proof

We first show by induction that (2.19) holds. Since $A_0=0$ and $\varPsi _0=d_{p+1}(x-x_0)$, it clearly holds for $k=0$. We now assume that inequality (2.19) holds for $k \ge 0$ and prove it for $k+1$. From (2.22), the induction assumption $\varPsi _k^*\ge A_kF(x_k)$, and the subgradient inequality, we obtain

$$\begin{aligned} \varPsi _{k+1}^*&= \min _{x\in {\textrm{dom}}\psi } \left\{ {}\varPsi _k(x)+a_{k+1}[\ell _{T_k}(x)+\psi (x)]{}\right\} \\&\ge \min _{x\in {\textrm{dom}}\psi } \left\{ {}\varPsi _k^*+\sigma _p \Vert x-\upsilon _k\Vert ^{p+1}+a_{k+1}[\ell _{T_k}(x)+\psi (x)]{}\right\} \\&\ge \min _{x\in {\textrm{dom}}\psi } \left\{ {}A_k F(x_k)+a_{k+1}[\ell _{T_k}(x)+\psi (x)]+\sigma _p \Vert x-\upsilon _k\Vert ^{p+1}{}\right\} \\&\ge \min _{x\in {\textrm{dom}}\psi } \left\{ A_k F(x_k)+a_{k+1}[f(T_k)+\left\langle {}\nabla f(T_k)+g_{k+1}{},{}x-T_k{}\right\rangle +\psi (T_k)]\right. \\ {}&\quad \left. +\sigma _p \Vert x-\upsilon _k\Vert ^{p+1}\right\} \\&= \min _{x\in {\textrm{dom}}\psi } \left\{ A_{k+1}F(T_k)+\left\langle {}\nabla f(T_k)+g_{k+1}{},{}a_{k+1}(x-T_k)+A_k(x_k-T_k){}\right\rangle \right. \\ {}&\quad \left. +\sigma _p \Vert x-\upsilon _k\Vert ^{p+1}\right\} \\&\ge \min _{x\in {\textrm{dom}}\psi } \left\{ A_{k+1}F(T_k)+\left\langle {}\nabla f(T_k)+g_{k+1}{},{}a_{k+1}(x-\upsilon _k)+A_{k+1}(y_k-T_k){}\right\rangle \right. \\ {}&\quad \left. + \sigma _p\Vert x-\upsilon _k\Vert ^{p+1}\right\} , \end{aligned}$$

with $\sigma _p=\tfrac{1}{p+1}\left( \tfrac{1}{2}\right) ^{p-1}$. For all $x\in {\textrm{dom}}\psi$, we have

$$\begin{aligned}&a_{k+1}\left\langle {}\nabla f(T_k)+g_{k+1}{},{}x-\upsilon _k{}\right\rangle +\tfrac{1}{p+1}\left( \tfrac{1}{2}\right) ^{p-1} \Vert x-\upsilon _k\Vert ^{p+1} \\&\quad \ge -\tfrac{p}{p+1} 2^{\tfrac{p-1}{p}} \left( a_{k+1}\Vert \nabla f(T_k)+g_{k+1}\Vert _*\right) ^{\tfrac{p+1}{p}}. \end{aligned}$$

It follows from (2.9) and $(T_k,g_{k+1})\in {\mathcal {A}}_p^H(y_k,\beta )$ that

$$\begin{aligned} \left\langle {}\nabla f(T_k)+g_{k+1}{},{}y_k-T_k{}\right\rangle \ge c_p \Vert \nabla f(T_k)+g_{k+1}\Vert _*^{\tfrac{p+1}{p}}. \end{aligned}$$

Combining the last three inequalities yields

2.24 $$\begin{aligned} \varPsi _{k+1}^*&\ge A_{k+1}F(T_k)+c_p A_{k+1} \Vert \nabla f(T_k)+g_{k+1}\Vert _*^{\tfrac{p+1}{p}}\nonumber \\&\quad -\tfrac{p}{p+1} 2^{\tfrac{p-1}{p}} \left( a_{k+1}\Vert \nabla f(T_k)+g_{k+1}\Vert _*\right) ^{\tfrac{p+1}{p}}\nonumber \\&= A_{k+1}F(T_k)+\left( c_p A_{k+1}-\tfrac{p}{p+1} 2^{\tfrac{p-1}{p}} a_{k+1}^{\tfrac{p+1}{p}}\right) \Vert \nabla f(T_k)+g_{k+1}\Vert _*^{\tfrac{p+1}{p}}\nonumber \\&\ge A_{k+1}F(T_k)+\left( c_p A_{k+1}-2a_{k+1}^{\tfrac{p+1}{p}}\right) \Vert \nabla f(T_k)+g_{k+1}\Vert _*^{\tfrac{p+1}{p}}. \end{aligned}$$

On the other hand, from (2.20), it can be deduced

$$\begin{aligned} \frac{(A_{k+1}-A_k)^{\tfrac{p+1}{p}}}{A_{k+1}}&=\frac{c_p}{2}\frac{\left( \left( \tfrac{k+1}{p+1}\right) ^{p+1}-\left( \tfrac{k}{p+1}\right) ^{p+1}\right) ^ {\tfrac{p+1}{p}}}{\left( \tfrac{k+1}{p+1}\right) ^{p+1}}\\&=\frac{c_p}{2} \left( \tfrac{k+1}{p+1}-\tfrac{k}{p+1}\left( 1-\tfrac{1}{k+1}\right) ^p\right) ^{\tfrac{p+1}{p}}\le \frac{c_p}{2}, \end{aligned}$$

leading to

$$\begin{aligned} a_k^{\tfrac{p+1}{p}}\le \tfrac{c_p}{2} A_{k+1}, \quad k\ge 0. \end{aligned}$$

Together with (2.24) and $f(T_k)\ge F(x_{k+1})$, this ensures $\varPsi _{k+1}^*\ge A_{k+1}F(x_{k+1})$, i.e., the assertion (i) holds. Invoking the inequalities (2.19) and (2.22), we come to

$$\begin{aligned} F(x_k)-F^*\le \tfrac{1}{A_k} d_{p+1}(x_0-x^*) = \left( \tfrac{2}{c_p}\right) ^p \left( \tfrac{p+1}{k}\right) ^{p+1} d_{p+1}(x_0-x^*), \end{aligned}$$

adjusting the inequality (2.23).

It follows from (2.19), (2.22), $F(x_k)-F^*\ge 0$, and $x=x^*$ that

$$\begin{aligned} d_{p+1}(x_0-x^*)&\ge -A_k F^*+\varPsi _k^*+\left( \tfrac{1}{2}\right) ^{p-1} d_{p+1}(\upsilon _k-x^*)\\&\ge (F(x_k)-F^*)+\left( \tfrac{1}{2}\right) ^{p-1}d_{p+1}(\upsilon _k-x^*), \end{aligned}$$

which leads to the assertion (iii). $\square$

## BiOPT: Bi-level OPTimization framework

As we have seen in the previous sections, solving the convex composite problem (2.1) by an inexact high-order proximal-point method involves two steps: (i) choosing a *p*th-order proximal-point method as an upper-level scheme; (ii) choosing a lower-level method for computing a point $(T,g)\in {\mathcal {A}}_p^H({\bar{x}}, \beta )$. This gives us two degrees of freedom in the strategy of finding a solution to the problem (2.1), which is why we call this framework *Bi-level OPTimization* (BiOPT). At the upper level, we do not need to impose any assumption on the objective $F(\cdot )$ apart from its convexity. At the lower-level method, we need some additional assumption on this objective function. Moreover, in the BiOPT setting, the complexity of a scheme leans on the complexity of both upper- and lower-level methods.

On the basis of the results of Sect. 2.1, the auxiliary problem (2.4) can be solved by applying one step of the *p*th-order tensor method. This demands the computation of *i*th ($i=1,\ldots ,p$) directional derivatives of function $f(\cdot )$ and the condition (2.13), which might not be practical in general. Therefore, we could try to apply a lower-order method to the auxiliary problem (2.4), which leads to an efficient implementation of the BiOPT framework. This is the main motivation of the following sections.

### Non-Euclidean composite gradient method

Let us assume that *k* is a fixed iteration of either Algorithm 1 or Algorithm 2, and we need to compute an *acceptable solution* $z_k$ of (2.4) satisfying (2.5). To do so, we introduce a non-Euclidean composite gradient method and analyze the convergence properties of the sequence $\{z_i\}_{i\ge 0}$ generated by this scheme, which satisfies in the limit inequality (2.5). Our main tool for such developments is the *relative smoothness condition* (see [9, 18] for more details and examples).

Notice that an acceptable solution of the auxiliary problem (2.4) requires that the function $\varphi _k:{\mathbb {E}}\rightarrow {\mathbb {R}}$ given by

3.1 $$\begin{aligned} \varphi _k(z)=f_{y_k,p}^H(z)+\psi (z), \quad \forall k\ge 0,\; z\in {\textrm{dom}}\psi \end{aligned}$$

be minimized approximately, delivering a point $y_k\in {\textrm{dom}}\psi$, satisfying the inequality (2.5). We here define $z_k^*=\mathop {\mathrm {arg\,min}}\limits _{z\in {\textrm{dom}}\psi } \varphi _k(z)$. Let us consider a simple example in which $f:{\mathbb {R}}\rightarrow {\mathbb {R}}$ with $f \equiv 0$ and $y_k=0$. Then, the function $f_{0,H}^2:{\mathbb {R}}\rightarrow {\mathbb {R}}$ defined as $f_{0,H}^2(z)=\tfrac{1}{3}|z|^3$ with $\nabla f_{0,H}^2(z)=|z|z$, which is not Lipschitz continuous. This shows that one cannot expect the Lipschitz smoothness of $f_{y_k,p}^H(\cdot )$ for $p\ge 2$. However, it can be shown that this function belongs to a wider class of functions called *relatively smooth*, which we describe next.

Let function $\rho :{\mathbb {E}}\rightarrow {\mathbb {R}}$ be closed, convex, and differentiable. We call it a *scaling function*. Now, the non-symmetric *Bregman* distance function $\beta _\rho :{\mathbb {E}}\times {\mathbb {E}}\rightarrow {\mathbb {R}}$ with respect to $\rho$ is given by

3.2 $$\begin{aligned} \beta _\rho (x,y)=\rho (y)-\rho (x)-\left\langle {}\nabla \rho (x){},{}y-x{}\right\rangle . \end{aligned}$$

For $x, y, z\in {\mathbb {E}}$, it is easy to see (e.g., the proof of Lemma 3 in [28]) that

3.3 $$\begin{aligned} \beta _\rho (x,z)-\beta _\rho (y,z)+\beta _\rho (y,x)=\left\langle {}\nabla \rho (y)-\nabla \rho (x){},{}z-x{}\right\rangle . \end{aligned}$$

For a convex function $h:{\mathbb {E}}\rightarrow {\mathbb {R}}$, we say that $h(\cdot )$ is *L*-*smooth relative to* $\rho (\cdot )$ if there exists a constant $L>0$ such that $(L\rho -h)(\cdot )$ is convex, and we call it $\mu$-*strongly convex relative to* $\rho (\cdot )$ if there exists $\mu >0$ such that $(h-\mu \rho )(\cdot )$ is convex; cf. [9, 18]. The constant $\kappa =\mu /L$ is called the *condition number* of $h(\cdot )$ relative to the scaling function $\rho (\cdot )$.

In the following lemma, we characterize the latter two conditions.

#### Lemma 3.1

[18, Proposition 1.1] The following assertions are equivalent:

(i) $h(\cdot )$ is $L$-smooth and $\mu$-strongly convex relative to the scaling function $\rho (\cdot )$;
(ii) $$ \mu \beta _\rho (x,y) \le h(x)-h(y)-\left\langle {}\nabla h(y){},{}x-y{}\right\rangle \le L\beta _\rho (x,y); $$
(iii) $$ \mu \left\langle {}\nabla \rho (y)-\nabla \rho (x){},{}y-x{}\right\rangle \le \left\langle {}\nabla h(y)-\nabla h(x){},{}y-x{}\right\rangle {} $$ $$\le {} L \langle \nabla \rho (y)- \nabla \rho (x),y-x \rangle $$;
(iv) $$ \mu \nabla ^2\rho (x) \preceq \nabla ^2h(x) \preceq L\nabla ^2\rho (x) $$.

Let us introduce the following assumptions on the minimization problem (3.1):

**(H1)**: $\rho (\cdot )$ is uniformly convex of degree $p+1$ and parameter $\sigma >0$, i.e., $\beta _\rho (x,y)\ge \tfrac{\sigma }{p+1} \Vert y-x\Vert ^{p+1}$;
**(H2)**: there exist constants $\mu , L> 0$ such that the function $f_{y_k,p}^H(\cdot )$ is *L*-smooth and $\mu$-strongly convex relative to the scaling function $\rho (\cdot )$.

Note that in (H2) we could introduce the parameters $\mu _{f_{y_k,p}^H}, L_{f_{y_k,p}^H}> 0$; however, for sake of simplicity we use $\mu , L> 0$. In this subsection, for the sake of generality, we assume the existence of the scaling function $\rho (\cdot )$ such that the conditions (H1)–(H2) hold; however, in Sect. 3.2 we introduce a specific scaling function satisfying (H1)–(H2).

We are in position now to develop a non-Euclidean composite gradient scheme for minimizing (3.1) based on the assumptions (H1)–(H2). For given $y_k,z_i\in {\textrm{dom}}\psi$ and $H, L>0$, we introduce the non-Euclidean composite gradient scheme

3.4 $$\begin{aligned} z_{i+1} = \mathop {\mathrm {arg\,min}}\limits _{z\in {\mathbb {E}}}\left\{ {}\left\langle {}\nabla f_{y_k,p}^H(z_i){},{}z-z_i{}\right\rangle +\psi (z)+2L \beta _\rho (z_i,z){}\right\} , \quad z_0=y_k, \end{aligned}$$

which is a first-order method and the point $z_k^*$ denotes the optimal solution of (3.4). Note that the first-order optimality conditions for (3.4) leads to the following variational principle

3.5 $$\begin{aligned} \left\langle {}\nabla f_{y_k,p}^H(z_i)+2L(\nabla \rho (z_{i+1})-\nabla \rho (z_i)){},{}z-z_{i+1}{}\right\rangle +\psi (z)\ge \psi (z_{i+1}). \end{aligned}$$

For the sequence $\{z_i\}_{i\ge 0}$ generated by the scheme (3.4), we next show the monotonicity of the sequence $\{\varphi _k(z_i)\}_{i\ge 0}$.

#### Lemma 3.2

(Non-Euclidean composite gradient inequalities) Let $\{z_i\}_{i\ge 0}$ be generated by the scheme (3.4). Then, for $z_0=y_k$, it holds that

3.6 $$\begin{aligned} \varphi _k(z_{i+1}) \le \varphi _k(z_i)-L \beta _\rho (z_i,z_{i+1}). \end{aligned}$$

Moreover, we have

3.7 $$\begin{aligned} \beta _{\rho }(z_{i+1},z) \le \vartheta ^{i+1} \beta _{\rho }(y_k,z) +\frac{1}{2L}\left( \frac{1-\vartheta ^{i+1}}{1-\vartheta }\right) \left( \varphi _k(z)-\varphi _k(z_{i+1})\right) , \end{aligned}$$

where $$\vartheta =1-\tfrac{\kappa }{2}$$.

#### Proof

Since $z_{i+1}$ is a solution of (3.4), it holds

$$\begin{aligned} \left\langle {}\nabla f_{y_k,p}^H(z_i){},{}z_{i+1}-z_i{}\right\rangle +\psi (z_{i+1})+2L \beta _\rho (z_i,z_{i+1}) \le \psi (z_i). \end{aligned}$$

Together with the *L*-smoothness of $f_{y_k,p}^H(\cdot )$ relative to $\rho (\cdot )$, this implies

$$\begin{aligned} f_{y_k,p}^H(z_{i+1})&\le f_{y_k,p}^H(z_i) + \left\langle {}\nabla f_{y_k,p}^H(z_i){},{}z_{i+1}-z_i{}\right\rangle +L \beta _\rho (z_i,z_{i+1})\\&\le f_{y_k,p}^H(z_i) + \psi (z_i)-\psi (z_{i+1})-L \beta _\rho (z_i,z_{i+1}), \end{aligned}$$

giving (3.6).

Setting $x=z_{i+1}$ and $y=z_i$ in the three point identity (3.3), applying the inequality (3.5), and using Lemma 3.1(b) it can be concluded that

3.8 $$\begin{aligned} \beta _{\rho }(z_{i+1},z) -\beta _{\rho }(z_i,z)&= \left\langle {}\nabla \rho (z_i)-\nabla \rho (z_{i+1}){},{}z-z_{i+1}{}\right\rangle -\beta _{\rho }(z_i,z_{i+1})\nonumber \\&\le \tfrac{1}{2L}\left[ \left\langle {}\nabla f_{y_k,p}^H(z_i){},{}z-z_{i+1}{}\right\rangle +\psi (z)-\psi (z_{i+1})\right] \nonumber \\&\quad -\beta _{\rho }(z_i,z_{i+1})\nonumber \\&= \tfrac{1}{2L}\left[ f_{y_k,p}^H(z_i)+\left\langle {}\nabla f_{y_k,p}^H(z_i){},{}z-z_i{}\right\rangle +\psi (z)\right] \nonumber \\&\quad - \tfrac{1}{2L}\left[ f_{y_k,p}^H(z_i)+\left\langle {}\nabla f_{y_k,p}^H(z_i){},{}z_{i+1}-z_i{}\right\rangle +\psi (z_{i+1})\right] \nonumber \\&\quad -\beta _{\rho }(z_i,z_{i+1})\nonumber \\&\le \tfrac{1}{2L}\left[ f_{y_k,p}^H(z_i)+\left\langle {}\nabla f_{y_k,p}^H(z_i){},{}z-z_i{}\right\rangle +\psi (z)-\varphi _k(z_{i+1})\right] \nonumber \\&\le \tfrac{1}{2L}\left[ \varphi _k(z)-\varphi _k(z_{i+1})-\mu \beta _{\rho }(z_i,z)\right] . \end{aligned}$$

Accordingly, since $\varphi _k(z_{i+1})\le \varphi _k(z_i)$ for $i\in {\mathbb {N}}$, we get

$$\begin{aligned} \beta _{\rho }(z_{i+1},z)&\le \vartheta \beta _{\rho }(z_i,z) +\tfrac{1}{2L}\left( \varphi _k(z)-\varphi _k(z_{i+1})\right) \\&\le \vartheta ^2 \beta _{\rho }(z_{i-1},z) +\tfrac{1}{2L}\left( 1+\vartheta \right) \left( \varphi _k(z)-\varphi _k(z_{i+1})\right) \\&\le \cdots \le \vartheta ^{i+1} \beta _{\rho }(y_k,z) +\tfrac{1}{2L} \left( \sum _{j=0}^i \vartheta ^j\right) \left( \varphi _k(z)-\varphi _k(z_{i+1})\right) , \end{aligned}$$

justifying the inequality (3.7). $\square$

In summary, we come to the following non-Euclidean composite gradient algorithm.
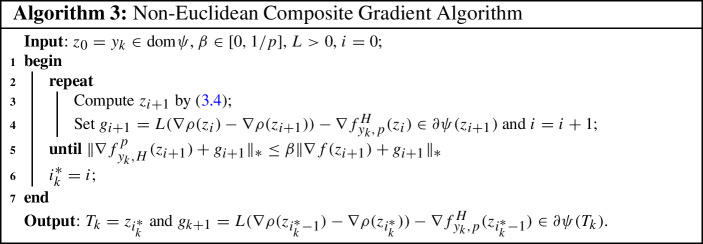


We now assume that the auxiliary problem (3.4) can be solved exactly. For the sequence $\{z_i\}_{i\ge 0}$ given by (3.4), we will stop the scheme as soon as $\Vert \nabla f_{y_k, H}^p(z_{i+1})+g_{i+1}\Vert _*\le \beta \Vert \nabla f(z_{i+1})+g_{i+1}\Vert _*$ holds, and then we set $z_k=z_{i+1}$. In the remainder of this section, we show that this stopping criterion holds for *i* large enough.

Setting $z=y_k$ in the inequality (3.7), it follows the $(p+1)$-uniform convexity of $\rho (\cdot )$ with parameter $\sigma >0$ and $\vartheta =1-\tfrac{\kappa }{2}\in (0,1)$ that

$$\begin{aligned} \Vert y_k-z_{i+1}\Vert ^{p+1}&\le \tfrac{p+1}{\sigma } \beta _{\rho }(z_{i+1},y_k) \le \tfrac{p+1}{2\sigma L} \left( \frac{1-\vartheta ^{i+1}}{1-\vartheta }\right) \left( \varphi _k(y_k)-\varphi _k(z_{i+1})\right) \\&\le \tfrac{p+1}{2\sigma L}\left( \frac{1-\vartheta ^{i+1}}{1-\vartheta }\right) \left( \varphi _k(y_k)-\inf \varphi _k\right) \le \tfrac{p+1}{\sigma \mu }\left( F(y_k)-F^*\right) \\&<+\infty ,\quad \forall i\in {\mathbb {N}}, \end{aligned}$$

Let us define the bounded convex set

3.9 $$\begin{aligned} {\mathcal {L}}_k(y_k, \varDelta _k)&= \left\{ {}z\in {\mathbb {E}}: \Vert y_k-z\Vert \le \varDelta _k, \varphi _k(z)\le \varphi _k(y_k){}\right\} ,\nonumber \\ \varDelta _k&=\left( \tfrac{p+1}{\sigma \mu }\left( F(y_k)-F^*\right) \right) ^{1/(p+1)}, \end{aligned}$$

i.e., $$\{z_i\}_{i\ge 0} \subseteq {\mathcal {L}}_k(y_k, \varDelta _k)$$.

The next results shows that the sequence $\{{\textrm{dist}}(0,\partial \varphi _k(z_i))\}_{i\ge 0}$ vanishes, for $\{z_i\}_{i\ge 0}$ generated by Algorithm 3. For doing so, we also require that

**(H3)**: $\Vert \nabla ^2\rho (\cdot )\Vert \le {\overline{L}}$ on the set ${\mathcal {L}}_k(y_k, \varDelta _k)$ with ${\overline{L}}>0$.

#### Lemma 3.3

(Subsequential convergence) Let $\{z_i\}_{i\ge 0}$ be generated by Algorithm 3. If (H1)–(H3) hold, then

3.10 $$\begin{aligned} \varphi _k(z_i)-\varphi _k(z_{i+1}) \ge C\Vert {\mathcal {G}}_{i+1}\Vert _*^{p+1}, \quad C=\tfrac{L\sigma }{(p+1)(L-\mu )^{p+1} {\overline{L}}^{p+1}}, \end{aligned}$$

where

3.11 $$\begin{aligned} {\mathcal {G}}_{i+1}&= \nabla f_{y_k,p}^H(z_{i+1})+g_{i+1},\nonumber \\ g_{i+1}&=L(\nabla \rho (z_i)-\nabla \rho (z_{i+1}))-\nabla f_{y_k,p}^H(z_i)\in \partial \psi (z_{i+1}). \end{aligned}$$

This consequently implies

3.12 $$\begin{aligned} \lim _{i\rightarrow +\infty } {\textrm{dist}}(0,\partial \varphi _k(z_{i+1})) = 0. \end{aligned}$$

#### Proof

Writing the first-order optimality conditions for the minimization problem (3.4), there exists $g_{i+1}\in \partial \psi (z_{i+1})$ such that

$$\begin{aligned} \nabla f_{y_k,p}^H(z_i)+g_{i+1}+L(\nabla \rho (z_{i+1})-\nabla \rho (z_i))=0, \end{aligned}$$

leading to

$$\begin{aligned} g_{i+1}=L(\nabla \rho (z_i)-\nabla \rho (z_{i+1}))-\nabla f_{y_k,p}^H(z_i). \end{aligned}$$

In light of the convexity of $f_{y_k,p}^H(\cdot )$ and $\psi (\cdot )$, we obtain $\partial \varphi _{k} (\cdot ) = \nabla f_{y_k,p}^H(\cdot )+\partial \psi (\cdot )$, i.e.,

$$\begin{aligned} {\mathcal {G}}_{i+1} = \nabla f_{y_k,p}^H(z_{i+1})+g_{i+1}\in \partial \varphi _k (z_{i+1}). \end{aligned}$$

On the bounded set ${\mathcal {L}}_k(y_k, \varDelta _k)$, it holds that

$$\begin{aligned} -{\mathcal {G}}_{i+1}&= L (\nabla \rho (z_{i+1})-\nabla \rho (z_i)) - (\nabla f_{y_k,p}^H(z_{i+1})-\nabla f_{y_k,p}^H(z_i))\\&= \int _0^1 [(L \nabla ^2\rho -\nabla ^2 f_{y_k,p}^H)(z_i+\tau (z_{i+1}-z_i))](z_{i+1}-z_i)d\tau . \end{aligned}$$

Let us define

$$\begin{aligned} B&=\left( L\nabla ^2 \rho (z)\right) ^{-\tfrac{1}{2}} \nabla ^2f_{y_k,p}^H(z) \left( L\nabla ^2 \rho (z)\right) ^{-\tfrac{1}{2}}, \\ z&= z_i+\tau (z_{i+1}-z_i),\quad \tau \in [0,1], \end{aligned}$$

which clearly implies

$$\begin{aligned}&\left( L \nabla ^2\rho (z)-\nabla ^2 f_{y_k,p}^H(z)\right) ^2= \left( \left( L\nabla ^2 \rho (z)\right) ^{\tfrac{1}{2}}(I-B)\left( L\nabla ^2 \rho (z)\right) ^{\tfrac{1}{2}}\right) ^2\\&= \left( L\nabla ^2 \rho (z)\right) ^{\tfrac{1}{2}}(I-B)\left( L\nabla ^2 \rho (z)\right) (I-B) \left( L\nabla ^2 \rho (z)\right) ^{\tfrac{1}{2}}. \end{aligned}$$

Together with (H2), (H3), and Lemma 3.1(iv), this leads to

3.13 $$\begin{aligned}&\Vert (L \nabla ^2\rho (z)-\nabla ^2 f_{y_k,p}^H(z))h\Vert ^2\nonumber \\&\quad = \left\langle {}[L \nabla ^2\rho (z)-\nabla ^2 f_{y_k,p}^H(z)]^2h{},{}h{}\right\rangle \nonumber \\&\quad =\left\langle {}\left( L\nabla ^2 \rho (z)\right) ^{\tfrac{1}{2}}(I-B)\left( L\nabla ^2 \rho (z)\right) (I-B) \left( L\nabla ^2 \rho (z)\right) ^{\tfrac{1}{2}}h{},{}h{}\right\rangle \nonumber \\&\quad =\left\langle {}(I-B) \left( L\nabla ^2 \rho (z)\right) ^{\tfrac{1}{2}}h{},{}\left( L\nabla ^2 \rho (z)\right) (I-B)\left( L\nabla ^2 \rho (z)\right) ^{\tfrac{1}{2}}h{}\right\rangle \nonumber \\&\quad \le L{\overline{L}} \left\langle {}(I-B)^2 \left( L\nabla ^2 \rho (z)\right) ^{\tfrac{1}{2}}h{},{}\left( L\nabla ^2 \rho (z)\right) ^{\tfrac{1}{2}}h{}\right\rangle \nonumber \\&\quad \le L{\overline{L}} \left( 1-\tfrac{\mu }{L}\right) ^2 \left\langle {}\left( L\nabla ^2 \rho (z)\right) ^{\tfrac{1}{2}}h{},{}\left( L\nabla ^2 \rho (z)\right) ^{\tfrac{1}{2}}h{}\right\rangle \nonumber \\&\quad \le L^2{\overline{L}}^2 \left( 1-\tfrac{\mu }{L}\right) ^2 \Vert h\Vert ^2. \end{aligned}$$

This and (H1) yield

$$\begin{aligned} \Vert {\mathcal {G}}_{i+1}\Vert _*&\le \Vert (L \nabla ^2\rho -\nabla ^2 f_{y_k,p}^H)(z)\Vert \Vert z_{i+1}-z_i\Vert \\&\le (L-\mu ) {\overline{L}} \Vert z_{i+1}-z_i\Vert \le (L-\mu ) {\overline{L}} \left( \tfrac{p+1}{\sigma } \beta _\rho (z_i,z_{i+1})\right) ^{1/(p+1)}. \end{aligned}$$

Thus, it can be concluded from (3.6) that

$$\begin{aligned} \varphi _k(z_i)- \varphi _k(z_{i+1}) \ge L \beta _\rho (z_i,z_{i+1})\ge \tfrac{L\sigma }{(p+1)(L-\mu )^{p+1} {\overline{L}}^{p+1}} \Vert {\mathcal {G}}_{i+1}\Vert _*^{p+1}, \end{aligned}$$

giving (3.10). Thus, $C\sum \Vert {\mathcal {G}}_{i+1}\Vert _*^{p+1} \le \varphi _k(y_k)-\inf \varphi _k\le F(y_k)-F^*<+\infty$, i.e., $\lim _{i\rightarrow \infty }\Vert {\mathcal {G}}_{i+1}\Vert =0$. Together with the inequality ${\textrm{dist}}(0,\partial \varphi _k(z_{i+1}))\le \Vert {\mathcal {G}}_{i+1}\Vert$, this implies (3.12). $\square$

We now show the well-definedness and complexity of Algorithm 3 in the subsequent result.

#### Theorem 3.4

(Well-definedness of Algorithm 3) Let us assume that all conditions of Lemma 3.3 hold, let $\{z_i\}_{i\ge 0}$ be a sequence generated by Algorithm 3, and let

3.14 $$\begin{aligned} F(z_i)-F(x^*)\ge \varepsilon , \quad \forall i\ge 0, \end{aligned}$$

where $x^*$ is a minimizer of $F(\cdot )$ and $\varepsilon >0$ is the accuracy parameter. Moreover, assume that there exists a constant $D>0$ such that $\Vert z_i-x^*\Vert \le D$ for all $i\ge 0$. Then, for the subgradients

$$\begin{aligned} {\mathcal {G}}_{i_k^*}&= \nabla f_{y_k,p}^H(z_{i_k^*})+g_{k+1}\in \partial \varphi _k (z_{i_k^*}),\quad g_{k+1}\\&=L(\nabla \rho (z_{i_k^*-1})-\nabla \rho (z_{i_k^*}))-\nabla f_{y_k,p}^H(z_{i_k^*-1}) \in \partial \psi (z_{i_k^*}), \end{aligned}$$

and $z_{i_k^*}\in {\textrm{dom}}\psi$, the maximum number of iterations $i_k^*$ needed to guarantee the inequality

3.15 $$\begin{aligned} \Vert {\mathcal {G}}_{i_k^*}\Vert _* \le \beta \Vert \nabla f(z_{i_k^*})+g_{k+1}\Vert _* \end{aligned}$$

satisfies

3.16 $$\begin{aligned} i_k^*\le 1+ \tfrac{2(p+1)}{\kappa }\log \left( \tfrac{\tfrac{D}{\beta } \left( \tfrac{2L}{C}\beta _{\rho }(y_k,z_k^*)\right) ^{1/(p+1)}}{ \varepsilon } \right) , \end{aligned}$$

where *C* is defined in (3.10) and $\varepsilon >0$ is the accuracy parameter.

#### Proof

Combining the subgradient and Cauchy–Schwartz inequalities with $\Vert z_i-x^*\Vert \le D$, it can be deduced that

3.17 $$\begin{aligned} \Vert \nabla f(z_i)+g_i\Vert _*\ge \tfrac{F(z_i)-F^*}{\Vert z_i-x^*\Vert }\ge \tfrac{F(z_i)-F^*}{D}\ge \tfrac{\varepsilon }{D}, \end{aligned}$$

for any $g_i\in \partial \psi (z_i)$. From (3.10), there exists $C>0$ such that

$$\begin{aligned} \varphi _k(z_{i_k^*-1})-\varphi _k(z_k^*)\ge \varphi _k(z_{i_k^*-1})-\varphi _k(z_{i_k^*}) \ge C\Vert {\mathcal {G}}_{i_k^*}\Vert _*^{p+1}, \end{aligned}$$

for ${\mathcal {G}}_{i_k^*}=\nabla f_{y_k,p}^H(z_{i_k^*})+g_{k+1}\in \partial \varphi _k(z_{i_k^*})$ and $g_{k+1}\in \partial \psi (z_{i_k^*})$. Together with (3.7), this implies

$$\begin{aligned} \Vert {\mathcal {G}}_{i_k^*}\Vert _*&\le C^{-1/(p+1)} \left( \varphi _k(z_{i_k^*-1})-\varphi _k(z_k^*)\right) ^{1/(p+1)}\\&\le \left( \tfrac{2L}{C}\right) ^{1/(p+1)}\left( \left( 1-\tfrac{\kappa }{2}\right) ^{i_k^*-1} \beta _{\rho }(y_k,z_k^*) -\beta _{\rho }(z_{i_k^*-1},z_k^*)\right) ^{1/(p+1)}\\&\le \left( \tfrac{2L}{C}\beta _{\rho }(y_k,z_k^*)\right) ^{1/(p+1)} \left( 1-\tfrac{\kappa }{2}\right) ^{(i_k^*-1)/(p+1)}. \end{aligned}$$

Since $1-\tfrac{\kappa }{2}\in (0,1)$, for large enough $i_k^*$,

we have $\tfrac{\beta \varepsilon }{D}\le \left( \tfrac{2\,L}{C}\beta _{\rho }(y_k,z_k^*)\right) ^{1/(p+1)} \left( 1-\tfrac{\kappa }{2}\right) ^{(i_k^*-1)/(p+1)}$, i.e., the bound (3.16) is valid by (3.17) with $i={i_k^*}$. $\square$

### Bi-level high-order methods

In the BiOPT framework, we here consider Algorithm 2 using the *p*th-order proximal-point operator in the upper-level, and in the lower-level we solve the auxiliary problem by the high-order non-Euclidean composite gradient method described in Algorithm 3. As such, our proposed algorithm only needs the *p*th-order oracle for even *p* and the $(p-1)$th-order oracle for odd *p*, which attains the complexity of ${\mathcal {O}}(\varepsilon ^{-1/(p+1)})$.

In the remainder of this section, we set $p\ge 2$ and $q=\lfloor p/2\rfloor$. For $\xi >1$, let us define the function $\rho _{y_k,p}^H:{\mathbb {E}}\rightarrow {\mathbb {R}}$ given by

3.18 $$\begin{aligned} \rho _{y_k,p}^H(x)= \sum _{j=1}^{q} \tfrac{2}{(2j)!} D^{2j} f(y_k)[x-y_k]^{2j}+\frac{3H}{2} d_{p+1}(x-y_k), \end{aligned}$$

which we will remarkably show to be uniformly convex with degree $p+1$ and parameter $\sigma _{\rho _{y_k,p}^H}=2^{1-p}H$. For $p=3$, the scaling function $\rho _k(z)=\frac{1}{2}\left\langle {}\nabla ^2f(y_k)(z-y_k){},{}z-y_k{}\right\rangle +3M_4(f) d_4(z-y_k)$ has been suggested in [29], which is slightly different than ours for $p=3$. Owing to this foundation, we can show that the function $f_{y_k,p}^H(\cdot )$ is *L*-smooth relative to the scaling function $\rho _{y_k,p}^H(\cdot )$, which paws the way toward algorithmic developments. We begin next with showing the uniform convexity of $\rho _{y_k,p}^H(\cdot )$. To this end, we need the *p*th-order Taylor expansion of the function $f(\cdot )$ around $y\in {\textrm{dom}}f$ given by

3.19 $$\begin{aligned} f(x) = \varOmega _{y,p}(x)+\tfrac{1}{p!} \int _0^1 (1-\xi )^p D^{p+1} f(y+\xi (x-y))[x-y]^{p+1} d\xi , \end{aligned}$$

for $x\in {\textrm{dom}}f$ and $\varOmega _{y,p}(x)=f(y)+\sum _{k=1}^p \tfrac{1}{k!} D^k f(y)[x-y]^k$. It is not hard to show that

3.20 $$\begin{aligned} \nabla ^2f(x)\preceq \nabla ^2\varOmega _{y,p}(x)+\tfrac{M_{p+1}(f)}{(p-1)!} \Vert x-y\Vert ^{p-1} B, \end{aligned}$$

see [27, Theorem 1].

#### Theorem 3.5

(Uniform convexity and smoothness of $\rho _{y_k,p}^H(\cdot )$) For any $x-y_k\in {\mathbb {E}}$ and $\xi >1$, if $p\ge 2$ and $q=\lfloor p/2\rfloor$, then

3.21 $$\begin{aligned} - {\mathcal {M}}_{y_k,p}(x)\preceq \sum _{j=1}^{q} \tfrac{1}{(2j-1)!} D^{2j+1} f(y_k)[x-y_k]^{2j-1} \preceq {\mathcal {M}}_{y_k,p}(x), \end{aligned}$$

where

$$\begin{aligned} {\mathcal {M}}_{y_k,p}(x)= \sum _{j=1}^{q} \tfrac{1}{(2j-2)!} D^{2j} f(y_k)[x-y_k]^{2j-2}+\tfrac{M_{p+1}(f)}{(p-1)!}\Vert x-y_k\Vert ^{p-1}B. \end{aligned}$$

Moreover, for

3.22 $$\begin{aligned} H = \tfrac{4M_{p+1}(f)}{(p-1)!}, \end{aligned}$$

$\rho _{y_k,p}^H(\cdot )$ given in (3.18) is uniformly convex with degree $p+1$ and parameter $\sigma _{\rho _{y_k,p}^H}= 2^{1-p}H$.

#### Proof

Let us fix arbitrary directions $u,h=x-y_k\in {\mathbb {E}}$. Setting $y=y_k$, it follows from (3.20) that

$$\begin{aligned} 0&\le \left\langle {}\nabla ^2f(x)u{},{}u{}\right\rangle \le \left\langle {}\nabla ^2\varOmega _{y_k,p}(x)u{},{}u{}\right\rangle +\tfrac{M_{p+1}(f)}{(p-1)!}\Vert h\Vert ^{p-1} \Vert u\Vert ^2\\&=\left\langle {}\sum _{i=2}^p \tfrac{1}{(i-2)!} D^i f(y_k)[h]^{i-2}u{},{}u{}\right\rangle +\tfrac{M_{p+1}(f)}{(p-1)!}\Vert h\Vert ^{p-1} \Vert u\Vert ^2. \end{aligned}$$

Hence, splitting the sum into the odd and even terms, we come to

$$\begin{aligned}&-\left\langle {}\sum _{j=1}^{q} \tfrac{1}{(2j-2)!} D^{2j} f(y_k)[h]^{2j-2}u{},{}u{}\right\rangle -\tfrac{M_{p+1}(f)}{(p-1)!}\Vert h\Vert ^{p-1} \Vert u\Vert ^2\\&\quad \le \left\langle {}\sum _{j=1}^{q} \tfrac{1}{(2j-1)!} D^{2j+1} f(y_k)[h]^{2j-1} u{},{}u{}\right\rangle , \end{aligned}$$

leading to the left hand side of (3.21). Replacing *h* by $-h$, it holds that

$$\begin{aligned} \left\langle {}\sum _{j=1}^{q} \tfrac{1}{(2j-1)!} D^{2j+1} f(y_k)[h]^{2j-1} u{},{}u{}\right\rangle&\le \left\langle {}\sum _{j=1}^{q} \tfrac{1}{(2j-2)!} D^{2j} f(y_k)[h]^{2j-2}u{},{}u{}\right\rangle \\&\quad +\tfrac{M_{p+1}(f)}{(p-1)!}\Vert h\Vert ^{p-1} \Vert u\Vert ^2, \end{aligned}$$

giving the right-hand side of (3.21).

From the *p*th-order Taylor expansion of the function *f* at $y_k$, (3.20), (3.21), and (1.3), we obtain

3.23 $$\begin{aligned} 0&\preceq \nabla ^2 f(x) \preceq \sum _{i=2}^p \tfrac{1}{(i-2)!} D^i f(y_k)[h]^{i-2}+ \tfrac{1}{(p-1)!} M_{p+1}(f)\Vert h\Vert ^{p-1} B\nonumber \\&\preceq \sum _{j=1}^{q} \tfrac{2}{(2j-2)!} D^{2j} f(y_k)[h]^{2j-2}+ \tfrac{2}{(p-1)!} M_{p+1}(f)\Vert h\Vert ^{p-1} B\nonumber \\&\preceq \sum _{j=1}^{q} \tfrac{2}{(2j-2)!} D^{2j} f(y_k)[h]^{2j-2}+ \tfrac{2}{(p-1)!} M_{p+1}(f)\nabla ^2 d_{p+1}(h)\nonumber \\&\preceq \sum _{j=1}^{q} \tfrac{2}{(2j-2)!} D^{2j} f(y_k)[h]^{2j-2}+ \frac{H}{2}\nabla ^2 d_{p+1}(h), \end{aligned}$$

which implies the convexity of the term $\sum _{j=1}^{q} \tfrac{2}{(2j)!} D^{2j} f(y_k)[h]^{2j}+ \tfrac{H}{2} d_{p+1}(h)$. Together with the uniform convexity of $d_{p+1}(\cdot )$ with degree $p+1$ and parameter $2^{1-p}$, this implies the uniform convexity of $\rho _{y_k, H}(\cdot )$ with degree $p+1$ and parameter $\sigma _{\rho _{y_k,p}^H}=2^{1-p}H$. $\square$

Theorem 3.5 is clearly implies that the assumption (H1) is satisfied for the scaling function $\rho _{y_k,p}^H(\cdot )$ (3.18). We next show that $f_{y_k, H}^p(\cdot )$ is smooth and strongly convex relative this scaling function for $p=3$, which is inspired by [27].

#### Theorem 3.6

(Relative smoothness and strong convexity of $f_{y_k, H}^p(\cdot )$ for $p=3$) For $p=3$ and $\xi >1$, it holds

3.24 $$\begin{aligned} \max \left\{ {}\tfrac{1}{2} \left( 1+\tfrac{1}{\xi }\right) ,\tfrac{5+\xi }{6}{}\right\} \nabla ^2 \rho _{y_k, H}(x)&\succeq \nabla ^2 f_{y_k,H}^3(x) \nonumber \\&\succeq \min \left\{ {}\tfrac{1}{2} \left( 1-\tfrac{1}{\xi }\right) ,\tfrac{5-\xi }{6}{}\right\} \nabla ^2 \rho _{y_k, H}(x), \end{aligned}$$

In particular, for $\xi =2$, function $f_{y_k, H}^3:{\mathbb {E}}\rightarrow {\mathbb {R}}$ is $\tfrac{3}{4}$-smooth and $\tfrac{1}{4}$-strongly convex relative to $\rho _{y_k,p}^H(\cdot )$ defined in (3.18).

#### Proof

Following the proof of Theorem 3.5 for $p=3$, it is not hard to show that (3.21) is satisfied with

$$\begin{aligned} {\mathcal {M}}_{y_k,3}(x)= \tfrac{1}{\xi } \left\langle {}\nabla ^2 f(y_k)h{},{}h{}\right\rangle +\tfrac{\xi M_4(f)}{2}\Vert h\Vert ^2 B, \quad h=x-y_k, \quad \xi >1. \end{aligned}$$

From the third-order Taylor expansion of the function *f* at $y_k$, (3.20), (3.21), and (1.3), we obtain

$$\begin{aligned} \nabla ^2 f(x)&\preceq \sum _{k=2}^3 \tfrac{1}{(k-2)!} D^k f(y_k)[h]^{k-2}+ \tfrac{1}{2} M_4(f)\Vert h\Vert ^2 B\\&\preceq \left( 1+\tfrac{1}{\xi }\right) \nabla ^2 f(y_k)+ \tfrac{(1+\xi )}{2} M_4(f)\Vert h\Vert ^2 B\\&\preceq \left( 1+\tfrac{1}{\xi }\right) \nabla ^2 f(y_k)+ \tfrac{(1+\xi )}{2} M_4(f)\nabla ^2 d_4(h). \end{aligned}$$

In light of $f_{y_k,H}^3(\cdot )=f(\cdot )+H d_4(\cdot -y_k)$, $H=2M_4(f)$, and (3.22), we can write

$$\begin{aligned} \nabla ^2 f_{y_k,H}^3(x)&\preceq \left( 1+\tfrac{1}{\xi }\right) \nabla ^2 f(y_k) + \left[ 2M_4(f)+\tfrac{(1+\xi )}{2} M_4(f)\right] \nabla ^2 d_4(x-y_k)\\&\preceq \max \left\{ {}\tfrac{1}{2} \left( 1+\tfrac{1}{\xi }\right) ,\tfrac{5+\xi }{6}{}\right\} \nabla ^2 \rho _{y_k, H}(x). \end{aligned}$$

On the other hand, applying the third-order Taylor expansion (3.19) and (3.21) yield

$$\begin{aligned} \nabla ^2 f(x)&\succeq \sum _{k=2}^3 \tfrac{1}{(k-2)!} D^k f(y_k)- \tfrac{1}{2} M_4(f)\Vert x-y_k\Vert ^2 B\\&\succeq \left( 1-\tfrac{1}{\xi }\right) \nabla ^2 f(y_k)- \tfrac{(1-\xi )}{2} M_4(f)\Vert h\Vert ^2 B\\&\succeq \left( 1-\tfrac{1}{\xi }\right) \nabla ^2 f(y_k)- \tfrac{(1-\xi )}{2} M_4(f)\nabla ^2 d_4(h). \end{aligned}$$

We therefore have

$$\begin{aligned} \nabla ^2 f_{y_k,H}^3(x)&\succeq \left( 1-\tfrac{1}{\xi }\right) \nabla ^2 f(y_k) + \left[ 2M_4(f)+\tfrac{(1-\xi )}{2} M_4(f)\right] \nabla ^2 d_4(x-y_k)\\&\succeq \min \left\{ {}\tfrac{1}{2} \left( 1-\tfrac{1}{\xi }\right) ,\tfrac{5-\xi }{6}{}\right\} \nabla ^2 \rho _{y_k, H}(x), \end{aligned}$$

giving (3.24). Setting $\xi =2$ in (3.24) and invoking Lemma 3.1(iv), the claims are valid. $\square$

While Theorem 3.6 establishes the smoothness and strong convexity of $f_{y_k, H}^p(\cdot )$ relative to $\rho _{y_k,p}^H(\cdot )$ for $p=3$, the relative strong convexity is not true for other $p\ge 2$. As such, in the subsequent result for arbitrary $p\ge 2$, we show that $f_{y_k, H}^p(\cdot )$ is 1-smooth relative to $\rho _{y_k,p}^H(\cdot )$ and $f_{y_k, H}^p(\cdot )$ is uniformly convex with degree $p+1$ and parameter $\sigma _{f_{y_k, H}^p}=2^{1-p}H$.

#### Theorem 3.7

(Relative smoothness of $f_{y_k, H}^p(\cdot )$) Let $H\ge M_{p+1}(f)$ and let $p\ge 2$ and $q=\lfloor p/2\rfloor$. Then, the function $f_{y_k, H}^p:{\mathbb {E}}\rightarrow {\mathbb {R}}$ is 1-smooth relative to $\rho _{y_k,p}^H(\cdot )$ defined in (3.18). Moreover, the function $f_{y_k, H}^p(\cdot )$ is uniformly convex with degree $p+1$ and parameter $\sigma _{f_{y_k, H}^p}=2^{1-p}H$.

#### Proof

In light of $f_{y_k,p}^H(\cdot )=f(\cdot )+H d_{p+1}(\cdot -y_k)$ and (3.23), we can write

$$\begin{aligned} \nabla ^2 f_{y_k,p}^H(x) \preceq \sum _{j=1}^{q} \tfrac{2}{(2j-2)!} D^{2j} f(y_k)[h]^{2j-2}+ \frac{3H}{2}\nabla ^2 d_{p+1}(h)= \nabla ^2 \rho _{y_k, H}(x), \end{aligned}$$

implying 1-smooth of $f_{y_k, H}^p(\cdot )$ relative to $\rho _{y_k,p}^H(\cdot )$.

It follows from the convexity of *f* and the uniform convexity of $d_{p+1}(\cdot )$ with degree $p+1$ and parameter $\sigma _{d_{p+1}}=2^{1-p}$ that

$$\begin{aligned} f_{y_k, H}^p(z)&\ge f(x) +\left\langle {}\nabla f(x){},{}x-x{}\right\rangle \\&\quad + H\left( d_{p+1}(x)+\left\langle {}\nabla d_{p+1}(x){},{}z-x{}\right\rangle +\tfrac{1}{p+1} 2^{1-p} \Vert z-x\Vert ^{p+1} \right) \\&=f_{y_k, H}^p(x)+\left\langle {}\nabla f_{y_k, H}^p(x){},{}z-x{}\right\rangle +\tfrac{H}{p+1} 2^{1-p} \Vert z-x\Vert ^{p+1}, \end{aligned}$$

implying the uniform convexity of $f_{y_k, H}^p(\cdot )$ with degree $p+1$ and parameter $\sigma _{f_{y_k, H}^p}=2^{1-p}H$. $\square$

Since $\psi (\cdot )$ is convex and $f_{y_k, H}^p(\cdot )$ is uniformly convex with degree $p+1$ and parameter $\sigma _{f_{y_k, H}^p}=2^{1-p}H$, it is clear that $\varphi _k(\cdot )$ is uniformly convex with degree $p+1$ and parameter $\sigma _{\varphi _k}=2^{1-p}H$. This and Lemma 3.6 yield

$$\begin{aligned} \varphi _k(y_k)\ge \cdots \ge \varphi _k(z_i) \ge \varphi _k(z_{i+1})\ge \varphi _k(z_k^*)+\tfrac{\sigma _{\varphi _k}}{p+1} \Vert z_{i+1}-z_k^*\Vert ^{p+1}, \end{aligned}$$

leading to

$$\begin{aligned} \Vert z_{i+1}-z_k^*\Vert&\le \left( \tfrac{(p+1)!2^{p-3}}{p M_{p+1}(f)} \left( \varphi _k(y_k)-\varphi _k(z_k^*)\right) \right) ^{\tfrac{1}{p+1}}\\&\le \left( \tfrac{(p+1)!2^{p-3}}{p M_{p+1}(f)} \left( F(y_k)-F^*\right) \right) ^{\tfrac{1}{p+1}}. \end{aligned}$$

In the same way, we have

$$\begin{aligned} \varphi _k(y_k)\ge \varphi _k(z_k^*)+\tfrac{\sigma _{\varphi _k}}{p+1} \Vert y_k-z_k^*\Vert ^{p+1}, \end{aligned}$$

leading to

$$\begin{aligned} \Vert y_k-z_k^*\Vert&\le \left( \tfrac{(p+1)!2^{p-3}}{p M_{p+1}(f)} \left( \varphi _k(y_k)-\varphi _k(z_k^*)\right) \right) ^{\tfrac{1}{p+1}}\\&\le \left( \tfrac{(p+1)!2^{p-3}}{p M_{p+1}(f)} \left( F(y_k)-F^*\right) \right) ^{\tfrac{1}{p+1}}. \end{aligned}$$

Hence, we come to the inequality

3.25 $$\begin{aligned} \Vert z_{i+1}-y_k\Vert&\le \Vert z_{i+1}-z_k^*\Vert +\Vert z_k^*-y_k\Vert \le \overline{\varDelta }_k, \nonumber \\ \overline{\varDelta }_k&=\left( \tfrac{(p+1)!2^{2p-2}}{p M_{p+1}(f)} \left( F(y_k)-F^*\right) \right) ^{\tfrac{1}{p+1}}\quad \forall i\in {\mathbb {N}}. \end{aligned}$$

Next, we define the bounded convex set

3.26 $$\begin{aligned} {\mathcal {L}}_k(y_k, \overline{\varDelta }_k) = \left\{ {}z\in {\mathbb {E}}: \Vert y_k-z\Vert \le \overline{\varDelta }_k,\varphi _k(z)\le \varphi _k(y_k){}\right\} , \end{aligned}$$

i.e., $\{z_i\}_{i\ge 0} \subseteq {\mathcal {L}}_k(y_k, \overline{\varDelta }_k)$. We next show that the scaling function $\rho _{y_k,p}^H(\cdot )$ satisfies (H3).

#### Lemma 3.8

For any $x\in {\mathcal {L}}_k(y_k, \overline{\varDelta }_k)$ and $\xi >1$, if $p\ge 2$ and $q=\lfloor p/2\rfloor$, then

3.27 $$\begin{aligned} \Vert \nabla ^2 \rho _{y_k,p}^H(\cdot )\Vert \le {\overline{L}},\quad \overline{L}= M_4(f) \overline{\varDelta }_k^2+2M_2(f)+\left( \tfrac{2M_{p+1}}{(p-1)!}+\tfrac{3pH}{2}\right) \overline{\varDelta }_k^{p-1}, \end{aligned}$$

where $M_{2}(f)<+\infty$, $M_{4}(f)<+\infty$, and $M_{p+1}(f)<+\infty$ and on the set ${\mathcal {L}}_k(y_k, \overline{\varDelta }_k)$ with

$$\begin{aligned} \overline{\varDelta }_k=\left( \tfrac{(p+1)!2^{2p-2}}{p M_{p+1}(f)} \left( F(y_k)-F^*\right) \right) ^{\tfrac{1}{p+1}}. \end{aligned}$$

#### Proof

It follows from (3.19) that

$$\begin{aligned} \nabla ^2 f(y_k+h)&= \nabla ^2 f(y_k)+\sum _{i=3}^p \tfrac{1}{(i-2)!} D^i f(y_k)[h]^{i-2}+r_{p+1}(h),\\ \nabla ^2 f(y_k-h)&= \nabla ^2 f(y_k)+\sum _{i=3}^p \tfrac{(-1)^{i-2}}{(i-2)!} D^i f(y_k)[h]^{i-2}+r_{p+1}(-h), \end{aligned}$$

where

$$\begin{aligned} r_{p+1}(h)&=\tfrac{p+1}{(p-1)!} \int _0^1 (1-\xi )^p D^{p+1} f(y_k+\xi h)[h]^{p-1} d\xi ,\quad \Vert r_{p+1}(\pm h)\Vert \\&\le \tfrac{M_{p+1}(f)}{(p-1)!} \Vert h\Vert ^{p-1}. \end{aligned}$$

Summing up the latter identities, we come to

3.28 $$\begin{aligned} \nabla ^2 f(y_k+h)+\nabla ^2 f(y_k-h)-(r_{p+1}(h)+r_{p+1}(-h))= \sum _{j=1}^q \tfrac{2}{(2j-2)!} D^{2j} f(y_k)[h]^{2j-2}. \end{aligned}$$

Moreover, it holds that

$$\begin{aligned} \nabla ^2 f(y_k+h)&= \nabla ^2 f(y_k)+ D^3 f(y_k)[h]+r_4(h),\\ \nabla ^2 f(y_k-h)&= \nabla ^2 f(y_k)- D^3 f(y_k)[h]+r_4(-h), \end{aligned}$$

leading to

3.29 $$\begin{aligned} \Vert \nabla ^2 f(y_k+h)+\nabla ^2 f(y_k-h)-2\nabla ^2 f(y_k)\Vert \le r_4(h)+r_4(-h)\le M_4(f) \Vert h\Vert ^2.\nonumber \\ \end{aligned}$$

In light of (1.3), we have

$$\begin{aligned} \Vert \nabla ^2 \rho _{y_k, H}(x)\Vert&\le \left\| \sum _{k=1}^{q} \tfrac{2}{(2k-2)!} D^{2k} f(y_k)[h]^{2k-2} \right\| + \tfrac{3pH}{2}\Vert h\Vert ^{p-1}\\&\le \left\| \nabla ^2 f(y_k+h)+\nabla ^2 f(y_k-h)-(r_{p+1}(h)+r_{p+1}(-h)) \right\| \\&\quad + \tfrac{3pH}{2} \Vert h\Vert ^{p-1}\\&\le \Vert \nabla ^2 f(y_k+h)+\nabla ^2 f(y_k-h)-2\nabla ^2 f(y_k)\Vert \\&\quad +2\Vert \nabla ^2 f(y_k)\Vert +\tfrac{2M_{p+1}}{(p-1)!} \Vert h\Vert ^{p-1}+ \tfrac{3pH}{2}\Vert h\Vert ^{p-1}\\&\le M_4(f) \Vert h\Vert ^2+2M_2(f)+\tfrac{2M_{p+1}}{(p-1)!} \Vert h\Vert ^{p-1}+\tfrac{3pH}{2}\Vert h\Vert ^{p-1}. \end{aligned}$$

For $x\in {\mathcal {L}}_k(y_k, \overline{\varDelta }_k)$ and $h=x-y_k$, we come to

$$\begin{aligned} \Vert \nabla ^2 \rho _{y_k, H}(x)\Vert \le M_4(f) \overline{\varDelta }_k^2+2M_2(f)+\left( \tfrac{2M_{p+1}}{(p-1)!}+\tfrac{3pH}{2}\right) \overline{\varDelta }_k^{p-1}, \end{aligned}$$

establishing (3.27). $$\square $$

From now on and for sake of simplicity, we denote $\rho _{y_k,p}^H(\cdot )$ and $\sigma _{f_{y_k, H}^p}$ by $\rho _k(\cdot )$ and $\sigma _k$, respectively. In order to upper bound the Bregman term $\beta _{\rho _k}(\cdot ,\cdot )$, we next define the *norm-dominated scaling function* inspiring by [30, Definition 2], which will be needed in the remainder of this section.

#### Definition 3.9

The scaling function $\rho (\cdot )$ is called *norm-dominated* on the set $S\subseteq {\mathbb {E}}$ by some function $\theta _S:{\mathbb {R}}_+\rightarrow {\mathbb {R}}_+$ if $\theta _S(\cdot )$ is convex with $\theta _S(0)=0$ such that

3.30 $$\begin{aligned} \beta _\rho (x,y) \le \theta _S(\Vert x-y\Vert ), \end{aligned}$$

for all $x\in S$ and $y\in {\mathbb {E}}$.

We first verify the norm-dominatedness of the function $d_{p+1}(\cdot )$ over the set

$$\begin{aligned} B_R = \left\{ {}x\in {\mathbb {E}}| \Vert x\Vert \le R{}\right\} , \end{aligned}$$

for $$R>0$$.

#### Lemma 3.10

(Norm-dominatedness of the scaling function $d_{p+1}(\cdot )$) Let $p\ge 2$ and $q=\lfloor p/2\rfloor$. Then, the scaling function $d_{p+1}(\cdot )$ is norm-dominated on $B_{R}$ by the function

3.31 $$\begin{aligned} \widetilde{\theta }_{{\mathcal {L}}_k}(\tau ) = p2^{p-2} R^{p-1} \tau ^2+ \frac{2^{p-1}}{p+1} \tau ^{p+1}. \end{aligned}$$

#### Proof

From (1.3), it is cear that

$$\begin{aligned} \Vert \nabla ^2 d_{p+1}(x)\Vert = \Vert \Vert x\Vert ^{p-1} B+(p-1)\Vert x\Vert ^{p-3}\Vert \le p\Vert x\Vert ^{p-1} B. \end{aligned}$$

Together with the definition of Bregman distances, the inequality $\left( a^{\tfrac{1}{\theta }}+a^{\tfrac{1}{\theta }}\right) ^{\theta }\le 2^{\theta -1}(a+b)$ for $a,b\ge 0$ and $\theta \ge 1$, and $\tau =\Vert y-x\Vert$, this implies

$$\begin{aligned} \beta _{d_{p+1}}(x,y)&= d_{p+1}(y)-d_{p+1}(x)-\left\langle {}\nabla d_{p+1}(x){},{}y-x{}\right\rangle \\&=\int _0^1 (1-t) \nabla ^2 d_{p+1}(x+t (y-x))[y-x]^2 d t\\&\le p\int _0^1 (1-t) \Vert y-x\Vert ^2 \Vert x+t(y-x)\Vert ^{p-1} d t\\&\le p\int _0^1 (1-t) \Vert y-x\Vert ^2 (\Vert x\Vert +t\Vert y-x\Vert )^{p-1} d t\\&\le p2^{p-1} \tau ^2\int _0^1 (1-t) (R^{p-1}+t^{p-1}\tau ^{p-1}) d t\\&=p2^{p-2} R^{p-1}\tau ^2+\tfrac{2^{p-1}}{p+1} \tau ^{p+1}, \end{aligned}$$

giving (3.31). $$\square $$

In order to show the norm-dominatedness of the scaling function $\rho _k(\cdot )$, we also need the following technical lemma.

#### Lemma 3.11

Let $p\ge 2$ and $q=\lfloor p/2\rfloor$, and let the function $\widehat{\rho }_k:{\mathbb {E}}\rightarrow {\mathbb {R}}$ be defined by

$$\begin{aligned} \widehat{\rho }_k(x) = \sum _{j=1}^{q} \tfrac{2}{(2j)!} D^{2j} f(y_k)[x-y_k]^{2j}, \end{aligned}$$

then $\widehat{\rho }_k(x)$ is $\widehat{L}$-smooth over

$$\begin{aligned} B_{\overline{\varDelta }_k}=\left\{ {}z\in {\mathbb {E}}: \Vert y_k-z\Vert \le \overline{\varDelta }_k{}\right\} , \end{aligned}$$

with $$\widehat{L}=M_4(f) \overline{\varDelta }_k^2+2M_2(f)+\tfrac{2M_{p+1}(f)}{(p-1)!} \overline{\varDelta }_k^{p-1}$$ and $$\overline{\varDelta }_k=\Big (\tfrac{(p+1)!2^{2p-2}}{p M_{p+1}(f)} \big (F(y_k)-F^*\big )\Big )^{\tfrac{1}{p+1}}$$.

#### Proof

Setting $h=x-y_k$ and using $x\in B_{\overline{\varDelta }_k}$, (3.28) and (3.29), we come to

$$\begin{aligned} \Vert \nabla ^2 \rho _{y_k, H}(x)\Vert&\le \left\| \sum _{k=1}^{q} \tfrac{2}{(2k-2)!} D^{2k} f(y_k)[h]^{2k-2} \right\| \\&\le \left\| \nabla ^2 f(y_k+h)+\nabla ^2 f(y_k-h)-(r_{p+1}(h)+r_{p+1}(-h)) \right\| \\&\le \Vert \nabla ^2 f(y_k+h)+\nabla ^2 f(y_k-h)-2\nabla ^2 f(y_k)\Vert +2\Vert \nabla ^2 f(y_k)\Vert \\&\quad +\tfrac{2M_{p+1}(f)}{(p-1)!} \Vert h\Vert ^{p-1}\\&\le M_4(f) \Vert h\Vert ^2+2M_2(f)+\tfrac{2M_{p+1}(f)}{(p-1)!} \Vert h\Vert ^{p-1}\\&\le M_4(f) \overline{\varDelta }_k^2+2M_2(f)+\tfrac{2M_{p+1}(f)}{(p-1)!} \overline{\varDelta }_k^{p-1}, \end{aligned}$$

which guarantees ${\widehat{L}}$-smoothness of $\widehat{\rho }_k(x)$ on $B_{\overline{\varDelta }_k}$. $\square$

Invoking Lemma 3.11, we next show that the norm-dominatedness of $\rho _k(\cdot )$ on $B_{\overline{\varDelta }_k}$.

#### Lemma 3.12

(Norm-dominatedness of the scaling function $\rho _k(\cdot )$) Let $p\ge 2$ and $q=\lfloor p/2\rfloor$. Then, the function $\rho _k(\cdot )$ is norm-dominated over $B_{\overline{\varDelta }_k}$ with $\overline{\varDelta }_k=\left( \tfrac{(p+1)!2^{2p-2}}{p M_{p+1}(f)} \left( F(y_k)-F^*\right) \right) ^{\tfrac{1}{p+1}}$ by the function

3.32 $$\begin{aligned} \theta _{{\mathcal {L}}_k}(\tau ) = \frac{\widehat{L}+3p2^{p-2}\overline{\varDelta }_k^{p-1} H}{2}\tau ^2+\frac{3H2^{p-2}}{p+1} \tau ^{p+1}, \end{aligned}$$

where $$\tau \ge 0$$.

#### Proof

In light of the definition of $\rho _k(\cdot )$ and the $\widehat{L}$-smoothness of $\widehat{\rho }_k(\cdot )$, it can be concluded that

3.33 $$\begin{aligned} \beta _{\rho _k}(x,y)&= \widehat{\rho }_k(y)-\widehat{\rho }_k(x)-\left\langle {}\nabla \widehat{\rho }_k(x){},{}y-x{}\right\rangle \nonumber \\&\quad + \tfrac{3}{2}H \left( d_{p+1}(y-y_k)-d_{p+1}(x-y_k)-\left\langle {}\nabla d_{p+1}(x-y_k){},{}y-x{}\right\rangle \right) \nonumber \\&\le \tfrac{\widehat{L}}{2} \Vert x-y\Vert ^2+\tfrac{3}{2} H \beta _{d_{p+1}}(x-y_k,y-y_k). \end{aligned}$$

Invoking Lemmas 3.10 and 3.11, we come to

$$\begin{aligned} \beta _{\rho _k}(x,y)&\le \frac{\widehat{L}}{2} \Vert x-y\Vert ^2+\frac{3}{2} H \beta _{d_{p+1}}(x-y_k,y-y_k)\\&\le \frac{\widehat{L}}{2} \Vert x-y\Vert ^2+\frac{3}{2} H \left[ p2^{p-2} \overline{\varDelta }_k^{p-1} \Vert x-y\Vert ^2+ \frac{2^{p-1}}{p+1} \Vert x-y\Vert ^{p+1}\right] , \end{aligned}$$

adjusting (3.32) for $$\tau =\Vert x-y\Vert $$. $$\square $$

Motivated by the identity (3.22), in the remainder of this section, we have

3.34 $$\begin{aligned} \sigma _k=\sigma _{\rho _k}=\tfrac{2^{3-p} M_{p+1}(f)}{(p-1)!}. \end{aligned}$$

Additionally, in view of (2.20), we consider

3.35 $$\begin{aligned} \beta =\tfrac{1}{p},\quad A_k=\tfrac{(p-1)(p-1)!}{p2^{p+2} M_{p+1}(f)} \left( \tfrac{k}{p+1}\right) ^{p+1}, \quad a_{k+1}=A_{k+1}-A_k, \quad \textrm{for}\; k\ge 0. \end{aligned}$$

We now present our accelerated high-order method by combining all above-mentioned facts into Algorithm 2 leading to the following algorithm.
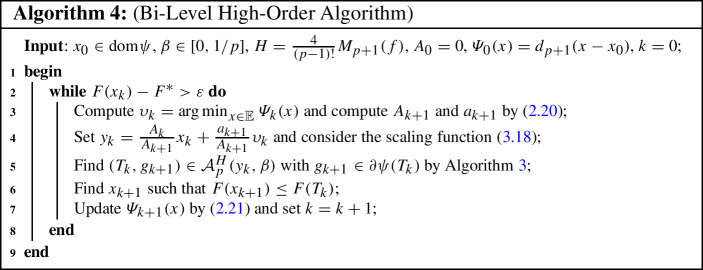


Since $L=1$ in our setting, the optimality conditions for the auxiliary problem (3.4) for the *p*th-order proximal-point operator is given by

$$\begin{aligned} \nabla f_{y_k, H}^p(z_i)+\partial \psi (z_{i+1})+2\left( \nabla \rho _k(z_{i+1})-\nabla \rho _k(z_i)\right) \ni 0, \end{aligned}$$

which should be solved exactly. We next translate this inclusion for convex constrained problem (2.2).

#### Example 3.13

We here revisit the convex constrained problem (2.2) and its unconstrained version (2.3) with $\psi (\cdot )=\delta _Q(\cdot )$. For $z_i\in {\mathbb {E}}$ and $L=1$, writing the first-order optimality conditions leads to

3.36 $$\begin{aligned} N_Q(z_{i+1}) \ni 2\left( \nabla \rho _k(z_i)-\nabla \rho _k(z_{i+1})\right) -\nabla f_{y_k, H}^p(z_i), \end{aligned}$$

where $\partial \psi (z_{i+1})=N_Q(z_{i+1})$ and therefore the *normal cone*

$$\begin{aligned} N_Q(x)=\left\{ \begin{array}{ll} \left\{ {}u\in {\mathbb {E}}: \left\langle {}u{},{}y-x{}\right\rangle \le 0,\; \forall y\in Q{}\right\} &{}\quad \textrm{if}\; x\in Q, \\ \emptyset &{}\quad \textrm{if}\; x\notin Q \end{array} \right. \end{aligned}$$

plays a crucial role for finding a solution of the auxiliary problem (3.4). As an example, let us consider the Euclidean ball $Q=\left\{ {}x\in {\mathbb {R}}^n : \Vert x\Vert \le \delta {}\right\}$ for which we have

$$\begin{aligned} N_Q(x)=\left\{ \begin{array}{ll} \left\{ {}\alpha x : \alpha >0{}\right\} &{}\quad \textrm{if}\; \Vert x\Vert =\delta , \\ \left\{ {}0{}\right\} &{}\quad \textrm{if}\; \Vert x\Vert <\delta . \end{array} \right. \end{aligned}$$

For $p=3$, our scaling function is given by

$$\begin{aligned} \rho _k(z)= \left\langle {}\nabla ^2f(y_k)(z-y_k){},{}z-y_k{}\right\rangle +\tfrac{3H}{8}\Vert z-y_k\Vert ^4. \end{aligned}$$

We now consider two cases: (i) $\Vert z_{i+1}\Vert <\delta$; (ii) $\Vert z_{i+1}\Vert =\delta$. In Case (i), we have

$$\begin{aligned}&\left( 4\nabla ^2f(y_k)(z_{i+1}-z_i)+3H\Vert z_{i+1}-y_k\Vert ^2B(z_{i+1}-y_k) -3H\Vert z_i-y_k\Vert ^2B(z_i-y_k)\right) \\&\quad -\nabla f_{y_k, H}^3(z_i)=0, \end{aligned}$$

with $$\nabla f_{y_k, H}^3(z_i)=\nabla f(z_i)+H\Vert z_i-y_k\Vert ^2(z_i-y_k)$$, i.e.,

$$\begin{aligned} \left[ 4\nabla ^2f(y_k)+3H\Vert z_{i+1}-y_k\Vert ^2B\right] (z_{i+1}-y_k)=b_i, \end{aligned}$$

for $b_i=\left[ 4\nabla ^2f(y_k)+H\Vert z_i-y_k\Vert ^2B\right] (z_i-y_k)+\nabla f_{y_k, H}^3(z_i)$. This consequently implies

$$\begin{aligned} z_{i+1}=y_k+\left[ 4\nabla ^2f(y_k)+3Hr^2B\right] ^{-1}b_i, \end{aligned}$$

where $r=\Vert z_{i+1}-y_k\Vert$ can be computed by solving the one-dimensional equation

$$\begin{aligned} r=\left\| \left[ 4\nabla ^2f(y_k)+3Hr^2B\right] ^{-1}b_i\right\| . \end{aligned}$$

In Case (ii) ($\Vert z_{i+1}\Vert =\delta$), there exists $\alpha >0$ such that

$$\begin{aligned} \left[ 4\nabla ^2f(y_k)+3H\Vert z_{i+1}-y_k\Vert ^2B\right] (z_{i+1}-y_k)-b_i=\alpha z_{i+1}, \end{aligned}$$

leading to

$$\begin{aligned} z_{i+1}=y_k+\left[ 4\nabla ^2f(y_k)+(3Hr^2-\alpha )B\right] ^{-1}(b_i+\alpha y_k), \end{aligned}$$

where $r=\Vert z_{i+1}-y_k\Vert$ and $\alpha$ are obtained by solving the system

$$\begin{aligned} \left\{ \begin{array}{l} r=\left\| \left[ 4\nabla ^2f(y_k)+(3Hr^2-\alpha )B\right] ^{-1}(b_i+\alpha y_k)\right\| ,\\ \delta = \left\| y_k+\left[ 4\nabla ^2f(y_k)+(3Hr^2-\alpha )B\right] ^{-1}(b_i+\alpha y_k)\right\| . \end{array} \right. \end{aligned}$$

Finally, we come to the solution

$$\begin{aligned} z_{i+1}=\left\{ \begin{array}{ll} y_k+\left[ 4\nabla ^2f(y_k)+3Hr^2B\right] ^{-1}b_i &{}\quad \textrm{if}\;\left\| y_k+\left[ 4\nabla ^2f(y_k)+3Hr^2B\right] ^{-1}b_i\right\| <\delta ,\\ y_k+\left[ 4\nabla ^2f(y_k)+(3Hr^2-\alpha )B\right] ^{-1}(b_i+\alpha y_k) &{}\quad \textrm{otherwise}, \end{array} \right. \end{aligned}$$

for the *r* and $\alpha$ computed by solving the above-mentioned nonlinear systems.$\square$

We now have all the ingredients to address the complexities of the upper and lower levels of Algorithm 4, which is the main result of this section. To this end, for the auxiliary minimization problem (3.4), we assume

3.37 $$\begin{aligned} R_0=\Vert x_0-x^*\Vert ,\quad D_0=\max _{z\in {\textrm{dom}}\psi } \left\{ {}\Vert z-x^*\Vert ~:~ F(z)\le F(x_0){}\right\} <+\infty . \end{aligned}$$

Let us set $S=\left\{ {}z\in {\textrm{dom}}\psi : \Vert z-x^*\Vert \le 2 R_0{}\right\}$ and assume

3.38 $$\begin{aligned} F_S = \sup _{z\in S} F(z)<+\infty . \end{aligned}$$

Let us note that the function $f_{y_k,p}^H(\cdot )$ is not strongly convex relative to the scaling function (3.18) for an arbitrary $p\ge 2$ (except for $p=3$, i.e., $q=1$), which implies that the results of Theorem 3.4 cannot be applied here. As such, for arbitrary *p*, we present the following result thanks to the uniform convexity of $f_{y_k,p}^H(\cdot )$ and $\rho _k(\cdot )$, for which we need the next technical lemma.

#### Lemma 3.14

Let $\varrho >1$, and let

3.39 $$\begin{aligned} \delta _i-\delta _{i+1}\ge \delta _i^\varrho \end{aligned}$$

for some integer $i\ge m \ge 0$. Then, $\delta _i<1$ and

3.40 $$\begin{aligned} \delta _i \le \frac{\delta _m}{[1+(\varrho -1)(i-m)\delta _m^{\varrho -1}]^{\tfrac{1}{\varrho -1}}} \le \frac{1}{[(\varrho -1)(i-m)]^{\tfrac{1}{\varrho -1}}}. \end{aligned}$$

#### Proof

The proof is a simple generalization of [13, Lemma 1.1]. $\square$

#### Theorem 3.15

(Complexity of Algorithm 4) Let us assume that all conditions of Theorem 3.4 hold, let $p\ge 2$ and $q=\lfloor p/2\rfloor$, let $M_2(f)<+\infty$, $M_4(f)<+\infty$, and $M_{p+1}(f)<+\infty$. Then,

(i) Algorithm 4 attains an $\varepsilon$-solution of the problem (2.1) in
  $$\begin{aligned} (2p+2) \left( \tfrac{3p M_{p+1}(f)}{(p-1)(p+1)(p-1)! \varepsilon }\right) ^{\tfrac{1}{p+1}} R_0 \end{aligned}$$
  iterations, for the accuracy parameter $\varepsilon >0$.
(ii) For $p=3$, the auxiliary problem (3.4) is approximately solved by Algorithm 3 in at most
  3.41 $$\begin{aligned} 1+ 24\log \left( \tfrac{\tfrac{D\overline{L}}{\beta } \left( \tfrac{\theta _{B_{D_1}}(D_1)}{M_4(f)}\right) ^{1/(p+1)}}{ \varepsilon } \right) \end{aligned}$$
  iterations with $D=D_1+\max \left\{ {}D_0,2R_0{}\right\}$, $B_{D_1}=\left\{ {}z\in {\mathbb {E}}: \Vert y_k-z\Vert \le D_1{}\right\}$ and
  $$\begin{aligned} D_1=\left( \tfrac{4}{(1-\vartheta )M_4(f)} (\max \left\{ {}F(x_0),F_S{}\right\} -F^*)\right) ^{\tfrac{1}{4}}, \quad \overline{L}= 2M_4(f) D_1^2+2M_2(f). \end{aligned}$$
(iii) For arbitrary *p*, the auxiliary problem (3.4) is approximately solved by Algorithm 3 in at most
  3.42 $$\begin{aligned} m+1+\frac{\tfrac{6\overline{L}}{p-1} \beta ^{1-p} \sigma _k^{-\tfrac{2}{p+1}} \overline{D}^{p-1} \overline{C}^{-\tfrac{p-1}{p+1}} (p+1)^{\tfrac{p+3}{p+1}}}{\varepsilon ^{p-1}} \end{aligned}$$
  iterations, where $\varphi _k(z_i)-\varphi _k(z_k^*)< \sigma _k^{-\tfrac{2}{p-1}} (p+1)^{\tfrac{2}{p-1}}(3\overline{L})^{\tfrac{p+1}{p-1}}$ for $i\ge m$ and
  $$\begin{aligned} \overline{D}=\left( \tfrac{(p+1)!2^{2p-2}}{pM_{p+1}(f)} (\max \left\{ {}F(x_0),F_S{}\right\} )\right) ^{\tfrac{1}{p+1}}+\max \left\{ {}D_0,2R_0{}\right\} . \end{aligned}$$

#### Proof

Assertion (i) follows directly from the inequality (2.23). To show (3.41) for $p=3$, we apply the results of Theorem 2.8. From Algorithm 4, we obtain

$$\begin{aligned} y_k= \tfrac{A_{k-1}}{A_k}x_k+\tfrac{a_k}{A_k}\upsilon _k, \quad \Vert \upsilon _k-x^*\Vert \le 2^{\tfrac{1}{2}} \Vert x_0-x^*\Vert , \end{aligned}$$

leading to

3.43 $$\begin{aligned} \Vert y_k-x^*\Vert&\le \tfrac{A_k}{A_{k+1}} \Vert x_k-x^*\Vert + \tfrac{a_{k+1}}{A_{k+1}} \Vert \upsilon _k-x^*\Vert \nonumber \\&\le \tfrac{A_k+a_{k+1}}{A_{k+1}} \max \left\{ {}\Vert x_k-x^*\Vert ,\Vert \upsilon _k-x^*\Vert {}\right\} \nonumber \\&\le \max \left\{ {}D_0,2^{\tfrac{1}{2}}R_0{}\right\} \nonumber \\&\le \max \left\{ {}D_0,2R_0{}\right\} . \end{aligned}$$

Invoking Theorem 2.8(iii), it holds that

$$\begin{aligned} \Vert \upsilon _k-x^*\Vert \le 2^{\tfrac{1}{2}} \Vert x_0-x^*\Vert \le 2 R_0, \end{aligned}$$

i.e., $\upsilon _k\in S$. Together with the convexity of $\psi (\cdot )$ and the monotonicity of the sequence $\{F(x_k)\}_{k\ge 0}$, this implies

3.44 $$\begin{aligned} F(y_k) \le \tfrac{A_k}{A_{k+1}} F(x_k) + \tfrac{a_{k+1}}{A_{k+1}} F(\upsilon _k) \le \tfrac{A_k}{A_{k+1}} F(x_0)+ \tfrac{a_{k+1}}{A_{k+1}} F_S \le \max \left\{ {}F(x_0),F_S{}\right\} . \end{aligned}$$

It follows from (3.6) that $F(z_i)\le \varphi _k(z_i)\le \varphi _k(z_{i-1})\le \cdots \le \varphi _k(y_k)=F(y_k)$. Setting $z=y_k$ in (3.7) and using and the latter inequality, we come to

$$\begin{aligned} \tfrac{(1-\vartheta )M_4(f)}{4} \Vert z_i-y_k\Vert ^4 \le F(y_k) -F(z_i) \le \max \left\{ {}F(x_0),F_S{}\right\} -F^*, \end{aligned}$$

leading to $\Vert z_i-y_k\Vert \le \left( \tfrac{4}{(1-\vartheta )M_4(f)} (\max \left\{ {}F(x_0),F_S{}\right\} -F^*)\right) ^{\tfrac{1}{4}}=D_1$. Hence, these inequalities lead to

$$\begin{aligned} \Vert z_i-x^*\Vert \le \Vert z_i-y_k\Vert + \Vert y_k-x^*\Vert \le D_1+\max \left\{ {}D_0,2R_0{}\right\} =D. \end{aligned}$$

For $p=3$, it is clear that all conditions of Theorem 3.4 are satisfied. On the other hand, from the definition $\theta _{B_{D_1}}(\cdot )$ given in (3.31), we obtain

$$\begin{aligned} \theta _R(\Vert z_k^*-y_k\Vert ) \le \theta _{B_{D_1}}(D_1). \end{aligned}$$

Then, from $L=\tfrac{3}{4}$, $\mu =\tfrac{1}{4}$, $\kappa =\tfrac{1}{3}$, the uniform convexity of $\rho _k(\cdot )$ with degree $\eta =4$ and parameter $\sigma _{\rho _k}=\tfrac{1}{2}M_4(f)$, $C=\tfrac{3M_4(f)}{2 {\overline{L}}^4}$, (3.31), $\overline{L}= 2M_4(f) D_1^2+2M_2(f)$ (see Lemma 3.11), and the proof of Theorem 3.4, we come to

$$\begin{aligned} i_k^*\le 1+ \tfrac{4}{-\log \left( 1-\tfrac{1}{6}\right) }\log \left( \tfrac{\tfrac{D}{\beta } \left( \tfrac{\overline{L}^4}{M_4(f)}\theta _{B_{D_1}}(D_1)\right) ^{\tfrac{1}{4}}}{ \varepsilon } \right) , \end{aligned}$$

which leads to (3.41) for $p=3$.

By Lemma 3.8, $\rho _k(\cdot )$ is $\widehat{L}$-smooth on $B_{\overline{\varDelta }_k}$ with $\widehat{L}=M_4(f) \overline{\varDelta }_k^2+2M_2(f)+\left( \tfrac{2M_{p+1}(f)}{(p-1)!}+\tfrac{3pH}{2}\right) \overline{\varDelta }_k^{p-1}$. Hence, from the convexity of $(\rho _k+f_{y_k,p}^H)(\cdot )$, and the uniform convexity of $\varphi _k(\cdot )$ with degree $p+1$ and parameter $\sigma _k=2^{1-p}H$, we obtain

$$\begin{aligned} \varphi _k(z_{i+1})&= \min _{z\in {\mathbb {E}}} \left\{ {}f_{y_k,p}^H(z_i)+\left\langle {}\nabla f_{y_k,p}^H(z_i){},{}z-z_i{}\right\rangle +\psi (z)+2\beta _{\rho _k}(z_i,z){}\right\} \\&\le \min _{z\in {\mathbb {E}}} \left\{ {}f_{y_k,p}^H(z)+\beta _{\rho _k}(z_i,z)+\psi (z)+2\beta _{\rho _k}(z_i,z){}\right\} \\&\le \min _{\alpha \in [0,1]} \left\{ {}\varphi _k(z)+3\beta _{\rho _k}(z_i,z) : z=z_i+\alpha (z_k^*-z_i){}\right\} \\&\le \min _{\alpha \in [0,1]} \left\{ {}\varphi _k(z)+\tfrac{3\overline{L}}{2} \Vert z-z_i\Vert ^2 ~:~ z=z_i+\alpha (z_k^*-z_i){}\right\} \\&\le \min _{\alpha \in [0,1]} \left\{ {}\varphi _k(z_i)- (\varphi _k(z_i)-\varphi _k(z_k^*))\alpha +\tfrac{3{\overline{L}}}{2} \Vert z_k^*-z_i\Vert ^2 \alpha ^2{}\right\} \\&\le \min _{\alpha \in [0,1]} \left\{ {}\varphi _k(z_i)- (\varphi _k(z_i)-\varphi _k(z_k^*))\alpha +\tfrac{3{\overline{L}}}{2} \left[ \tfrac{p+1}{\sigma _k}(\varphi _k(z_i)-\varphi _k(z_k^*))\right] ^{\tfrac{2}{p+1}} \alpha ^2{}\right\} . \end{aligned}$$

Minimizing the right hand-side of the last inequality with respect to $\alpha$ leads to the minimizer

$$\begin{aligned} \alpha ^*&=\min \left\{ {}\tfrac{1}{3\overline{L}} \left( \tfrac{\sigma _k}{p+1}\right) ^{\tfrac{2}{p+1}} (\varphi _k(z_i)-\varphi _k(z_k^*))^{\tfrac{p-1}{p+1}}, 1{}\right\} \\&= \min \left\{ {}\tfrac{\sigma _k}{(p+1)(3\overline{L})^{\tfrac{p+1}{2}}} (\varphi _k(z_i)-\varphi _k(z_k^*))^{\tfrac{p-1}{2}}, 1{}\right\} ^{\tfrac{2}{p+1}}\\&= \min \left\{ {}\alpha _{k,i}, 1{}\right\} ^{\tfrac{2}{p+1}}, \end{aligned}$$

where $\alpha _{k,i}=\sigma _k/[(p+1)(3\overline{L})^{\tfrac{p+1}{2}}] (\varphi _k(z_i)-\varphi _k(z_k^*))^{\tfrac{p-1}{2}}$. Let us consider two cases: (i) $\alpha _{k,i}<1$; (ii) $\alpha _{k,i}\ge 1$. In Case (i), we have $\alpha ^*=\alpha _{k,i}^{\tfrac{2}{p+1}}$, i.e.,

$$\begin{aligned} \varphi _k(z_{i+1})&\le \varphi _k(z_i)- \left( (\varphi _k(z_i)-\varphi _k(z_k^*)) -\tfrac{3{\overline{L}}}{2} \left[ \tfrac{p+1}{\sigma _k}(\varphi _k(z_i)-\varphi _k(z_k^*))\right] ^{\tfrac{2}{p+1}}\alpha _{k,i}^{\tfrac{2}{p+1}}\right) \alpha _{k,i}^{\tfrac{2}{p+1}}\\&= \varphi _k(z_i)- \left( (\varphi _k(z_i)-\varphi _k(z_k^*)) -\tfrac{1}{2} (\varphi _k(z_i)-\varphi _k(z_k^*))\right) \alpha _{k,i}^{\tfrac{2}{p+1}}\\&= \varphi _k(z_i)- \tfrac{1}{2} \alpha _{k,i}^{\tfrac{2}{p+1}} (\varphi _k(z_i)-\varphi _k(z_k^*)). \end{aligned}$$

In Case (ii), we have $\alpha _{i,k}\ge 1$, i.e., $\alpha ^*=1$ resulting to

$$\begin{aligned} \varphi _k(z_{i+1})&\le \varphi _k(z_i)- \left( (\varphi _k(z_i)-\varphi _k(z_k^*)) -\tfrac{3{\overline{L}}}{2} \left[ \tfrac{p+1}{\sigma _k}(\varphi _k(z_i)-\varphi _k(z_k^*))\right] ^{\tfrac{2}{p+1}}\right) \\&= \varphi _k(z_i)- \left( 1 -\tfrac{1}{2} \left[ \left( \tfrac{(p+1)(3\overline{L})^{\tfrac{p+1}{2}}}{\sigma _k}\right) (\varphi _k(z_i)-\varphi _k(z_k^*))^{\tfrac{1-p}{2}}\right] \right) \\&\quad \times (\varphi _k(z_i)-\varphi _k(z_k^*))\\&= \varphi _k(z_i)- \left( 1 -\tfrac{1}{2} \alpha _{k,i}^{-1} \right) (\varphi _k(z_i)-\varphi _k(z_k^*))\\&= \varphi _k(z_i)- \tfrac{1}{2} (\varphi _k(z_i)-\varphi _k(z_k^*)). \end{aligned}$$

Combining the both cases, we come to

$$\begin{aligned} \varphi _k(z_{i+1}) \le \varphi _k(z_i)- \tfrac{1}{2} \min \left\{ {}\alpha _{k,i}, 1{}\right\} ^{\tfrac{2}{p+1}} (\varphi _k(z_i)-\varphi _k(z_k^*)). \end{aligned}$$

Since $\{\varphi _k(z_i)\}_{k\ge 0}$ is decreasing, there exists $m\in {\mathbb {N}}$ such that $\alpha _{k,i}<1$ for all $i\ge m$, i.e., the latter inequality is translated to

$$\begin{aligned}&\varphi _k(z_{i+1})-\varphi _k(z_k^*) \le \varphi _k(z_i)-\varphi _k(z_k^*)\\&\quad - \left[ \left( \tfrac{1}{6\overline{L}}\right) ^{\tfrac{p+1}{p-1}} \left( \tfrac{\sigma _k}{p+1}\right) ^{\tfrac{2}{p-1}} (\varphi _k(z_i)-\varphi _k(z_k^*))\right] ^{\tfrac{p-1}{p+1}}(\varphi _k(z_i)-\varphi _k(z_k^*)), \end{aligned}$$

for $i\ge m$. Setting $\delta _k=\left( \tfrac{1}{6\overline{L}}\right) ^{\tfrac{p+1}{p-1}} \left( \tfrac{\sigma _k}{p+1}\right) ^{\tfrac{2}{p-1}} (\varphi _k(z_i)-\varphi _k(z_k^*))$, it holds that

$$\begin{aligned} \delta _i-\delta _{i+1}\ge \delta _i^{\tfrac{2p}{p+1}}, \end{aligned}$$

i.e., the inequality (3.39) is satisfied with $\varrho =\tfrac{2p}{p+1}$. Together with Lemma 3.14, this implies

3.45 $$\begin{aligned} \varphi _k(z_i)-\varphi _k(z_k^*)\le \sigma _k^{-\tfrac{2}{p-1}} (p-1)^{-\tfrac{p+1}{p-1}} (6\overline{L})^{\tfrac{p+1}{p-1}} (p+1)^{\tfrac{p+3}{p-1}} \left( \frac{1}{i-m}\right) ^{\tfrac{p+1}{p-1}}. \end{aligned}$$

On the other hand, following the proof of Lemma 3.3 and using 1-smoothness relative to $\rho _k(\cdot )$, (3.13), and the uniform convexity of $\rho _k$ with degree $p+1$ and parameter $\sigma _{\rho _k}$, we come to

$$\begin{aligned} \Vert {\mathcal {G}}_{i+1}\Vert _*\le \Vert (\nabla ^2 \rho _k-\nabla ^2 f_{y_k,p}^H)(z)\Vert \Vert z_{i+1}-z_i\Vert \le \overline{L} \left( \tfrac{p+1}{\sigma _{\rho _k}} \beta _{\rho _k}(z_i,z_{i+1})\right) ^{1/(p+1)}, \end{aligned}$$

Together with (3.6), this implies

$$\begin{aligned}&\varphi _k(z_i)- \varphi _k(z_{i+1}) \ge \beta _{\rho _k}(z_i,z_{i+1})\ge \tfrac{\sigma _{\rho _k}}{(p+1) {\overline{L}}^{p+1}} \Vert {\mathcal {G}}_{i+1}\Vert _*^{p+1}=\overline{C} \Vert {\mathcal {G}}_{i+1}\Vert _*^{p+1},\\&\overline{C}= \tfrac{\sigma _{\rho _k}}{(p+1) {\overline{L}}^{p+1}}, \end{aligned}$$

which consequently leads to

$$\begin{aligned} \overline{C}^{-\tfrac{1}{p+1}} \left( \varphi _k(z_{i_k^*-1})-\varphi _k(z_k^*)\right) ^{\tfrac{1}{p+1}}&\ge \overline{C}^{-\tfrac{1}{p+1}} \left( \varphi _k(z_{i_k^*-1})-\varphi _k(z_{i_k^*})\right) ^{\tfrac{1}{p+1}} \ge \Vert {\mathcal {G}}_{i_k^*}\Vert _*, \end{aligned}$$

with ${\mathcal {G}}_{i_k^*}=\nabla f_{y_k,p}^H(z_{i_k^*})-\nabla f_{y_k,p}^H(z_{i_k^*-1})+ \nabla \rho _k(z_{i_k^*-1})-\nabla \rho _k(z_{i_k^*})\in \partial \varphi _k(z_{i_k^*})$. Moreover, combining (3.25), (3.43), and (3.44) yields

$$\begin{aligned} \Vert z_i-x^*\Vert&\le \Vert z_i-y_k\Vert +\Vert y_k-x^*\Vert \\&\le \left( \tfrac{(p+1)!2^{2p-2}}{pM_{p+1}(f)} (\max \left\{ {}F(x_0),F_S{}\right\} )\right) ^{\tfrac{1}{p+1}}+\max \left\{ {}D_0,2R_0{}\right\} =\overline{D}. \end{aligned}$$

It follows from (3.45) that

$$\begin{aligned} \Vert {\mathcal {G}}_{i_k^*}\Vert _*&\le \overline{C}^{-\tfrac{1}{p+1}} \left( \varphi _k(z_{i_k^*-1})-\varphi _k(z_k^*)\right) ^{\tfrac{1}{p+1}}\\&\le \sigma _k^{-\tfrac{2}{p^2-1}} (p-1)^{-\tfrac{1}{p-1}} \overline{C}^{-\tfrac{1}{p+1}}(6\overline{L})^{\tfrac{1}{p-1}} (p+1)^{\tfrac{p+3}{p^2-1}} \left( \frac{1}{i_k^*-m-1}\right) ^{\tfrac{1}{p-1}}. \end{aligned}$$

Then, the inequality

$$\begin{aligned} \sigma _k^{-\tfrac{2}{p^2-1}} (p-1)^{-\tfrac{1}{p-1}} \overline{C}^{-\tfrac{1}{p+1}}(6\overline{L})^{\tfrac{1}{p-1}} (p+1)^{\tfrac{p+3}{p^2-1}} \left( \frac{1}{i_k^*-m-1}\right) ^{\tfrac{1}{p-1}} \ge \tfrac{\beta \varepsilon }{\overline{D}} \end{aligned}$$

gives (3.42). $$\square $$

Let us fix $q\ge 1$. Then, the function $f_{y_k,p}^H(\cdot )$ is *L*-smooth relative to the scaling function $\rho _k(\cdot )$ (3.18), which is the same for both $p=2q$ and $p=2q+1$. If *p* is even (i.e., $p=2q$), then Algorithm 4 is a 2*q*-order method (requiring the 2*q*-order oracle) and attains the complexity of ${\mathcal {O}}(\varepsilon ^{-1/(2q+1)})$, which worse than the optimal complexity ${\mathcal {O}}(\varepsilon ^{-2/(6q+1)})$. On the other hand, if *p* is odd ($p=2q+1$), then Algorithm 4 is again a 2*q*-order method (requiring the 2*q*-order oracle) obtaining the complexity of ${\mathcal {O}}(\varepsilon ^{-1/(2q+2)})$, which is also worse than the optimal complexity ${\mathcal {O}}(\varepsilon ^{-2/(6q+1)})$ except for $p=3$ that leads to the complexity ${\mathcal {O}}(\varepsilon ^{-(1/4)})$ overpassing the optimal complexity bound of second-order methods, i.e., ${\mathcal {O}}(\varepsilon ^{-(2/7)})$, as was known from in [29]. However, in the following example, we show that the complexity of our method can overpass the classical bounds for some structured class of problems. It is arguable that one may come up with algorithms with better complexity than (3.42) for finding the acceptable solution satisfying (2.5) for general *p* (e.g., with a more sophisticated methods or stronger assumptions); however, this out of scope of the current study and will be further investigated in our future work.

#### Example 3.16

Let us consider the vector $b\in {\mathbb {R}}^N$, the vectors $a_i\in {\mathbb {R}}^n$ and the univariate functions $f_i:{\mathbb {R}}\rightarrow {\mathbb {R}}$ that are four times continuously differentiable, for $i=1,\ldots ,N$. Then, we define the function $f:{\mathbb {R}}^n\rightarrow {\mathbb {R}}$ as

3.46 $$\begin{aligned} f(x)=\sum _{i=1}^N f_i(\left\langle {}a_i{},{}x{}\right\rangle -b_i). \end{aligned}$$

We are interested to apply Algorithm 4 with $p=4$ and $p=5$ to the problem (2.1) with this function $f(\cdot )$. In case of $p=5$, $q=\lfloor 5/2\rfloor =2$ and we need to handle the subproblem

$$\begin{aligned} z_{i+1} = \mathop {\mathrm {arg\,min}}\limits _{z\in {\mathbb {E}}}\left\{ {}\left\langle {}\nabla f_{y_k,H}^5(z_i){},{}z-z_i{}\right\rangle +\psi (z)+2L \beta _{\rho _k}(z_i,z){}\right\} , \end{aligned}$$

with

$$\begin{aligned} \rho _k(x)= \left\langle {}\nabla ^2 f(y_k)(x-y_k){},{}x-y_k{}\right\rangle + \tfrac{1}{12}D^4 f(y_k)[x-y_k]^4+\tfrac{3}{2} H d_6(x-y_k), \end{aligned}$$

which readily implies that our method requires fourth-order oracle of $f_i(\cdot )$, for $i=1,\ldots ,N$. Let us emphsize that Theorem 3.5 implies that the sacling function $\rho _k(\cdot )$ is convex, which is an interesting result even in one dimension and with $N=1$, i.e.,

$$\begin{aligned} 2f''(y_k) + f^{iv}(y_k)h^2+\tfrac{15}{2} H|x-y_k|^4 \succeq 0. \end{aligned}$$

In the same way, for $p=4$, we need fourth-order oracle of $f_i(\cdot )$, for $i=1,\ldots ,N$. Moreover, Theorem 3.15 ensures that the sequence generated by Algorithm 4 attains the complexity ${\mathcal {O}}(\varepsilon ^{-1/5})$ for $p=4$ and ${\mathcal {O}}(\varepsilon ^{-1/6})$ for $p=5$, which are worse that the optimal complexity ${\mathcal {O}}(\varepsilon ^{-2/13})$, for the accuracy parameter $\varepsilon$. On the other hand, setting $h=x-y_k$, it holds that

$$\begin{aligned} \left\langle {}\nabla ^2 f(y_k)h{},{}h{}\right\rangle&= \sum _{i=1}^N \nabla ^2 f_i(\left\langle {}a_i{},{}y_k{}\right\rangle -b_i) \left\langle {}a_i{},{}h{}\right\rangle ^2,\\ D^4 f(y_k)[h]^4&= \sum _{i=1}^N \nabla ^4 f_i(\left\langle {}a_i{},{}y_k{}\right\rangle -b_i) \left\langle {}a_i{},{}h{}\right\rangle ^4. \end{aligned}$$

Let us particularly verify these terms for $f_i(x)=-\log (x)$ ($i=1,\ldots ,N$) for $x\in (0,+\infty )$, which consequently leads to

$$\begin{aligned} \nabla ^2f_i(x)=\frac{1}{x^2}, \quad \nabla ^4f_i(x)=\frac{6}{x^4}=6\left( \nabla ^2f_i(x)\right) ^2, \end{aligned}$$

i.e.,

$$\begin{aligned} D^4 f(y_k)[h]^4 = 6\sum _{i=1}^N \left( \nabla ^2f_i(\left\langle {}a_i{},{}y_k{}\right\rangle -b_i)\right) ^2 \left\langle {}a_i{},{}h{}\right\rangle ^4. \end{aligned}$$

Thus, in this case, the implementation of Algorithm 4 with $p=4$ and $p=5$ only requires the second-order oracle of $f_i(\cdot )$ ($i=1,\ldots ,N$) and the first-order oracle of $\psi (\cdot )$. Therefore, we end up with a second-order method with the complexity of ${\mathcal {O}}(\varepsilon ^{-1/5})$ for $p=4$ and ${\mathcal {O}}(\varepsilon ^{-1/6})$ for $p=5$, which are much faster than the second-order methods optimal bound ${\mathcal {O}}(\varepsilon ^{-2/7})$; however, choosing the odd order $p=5$, Algorithm 4 attains the better complexity. $\square$

## Conclusion

In this paper, we suggest a bi-level optimization (BiOPT), a novel framework for solving convex composite minimization problems, which is a generalization of the BLUM framework given in [29] and involves two levels of methodologies. In the upper level, we only assume the convexity of the objective function and design some upper-level scheme using a high-order proximal-point iterations with arbitrary order. On the other hand, in the lower level, we need to solve the proximal-point auxiliary problem inexactly by some lower-level scheme. In this step, we require some more properties of the objective function for developing efficient algorithms providing acceptable solutions for this auxiliary problem at a reasonable computational cost. The overall complexity of the method will be the product of the complexities in both levels.

We here develop the basic *p*th-order inexact proximal-point method and its acceleration using the estimation sequence technique that, respectively, achieve the convergence rate ${\mathcal {O}}(k^{-p})$ and ${\mathcal {O}}(k^{-(p+1)})$ for the iteration counter *k*. Assuming the *L*-smoothness and $\mu$-strong convexity of the differentiable part of the proximal-point objective relative to some scaling function (for $L, \mu >0$), we design a non-Euclidean composite gradient method to inexactly solve the proximal-point problem. It turns out that this method attains the complexity ${\mathcal {O}}(\log \tfrac{1}{\varepsilon })$, for the accuracy parameter $\varepsilon >0$.

In the BiOPT framework, we apply the accelerated *p*th-order proximal-point algorithm in the upper level, introduce a new high-order scaling function and show that the differentiable part of the auxiliary objective is smooth relative to this function, and solve the auxiliary problem by a non-Euclidean composite gradient method in the lower level with the complexity of ${\mathcal {O}}\left( \log \tfrac{1}{\varepsilon }\right)$. We consequently come to a bi-level high-order method with the complexity of ${\mathcal {O}}(\varepsilon ^{-1/(p+1)})$, which overpasses the classical complexity bound of second-order methods for $p=3$, as was known from [29]. In general, for $p=2$ and $p\ge 3$, the complexity of our bi-level method is sub-optimal; however, we showed that for some class of structured problems it can overpass the optimal complexity bound ${\mathcal {O}}(\varepsilon ^{-2/(3p+1)})$. Overall, the BiOPT framework paves the way toward methodologies using the *p*th-order proximal-point operator in the upper level and requiring lower-order oracle than *p* in the lower level. Therefore, owing to this framework, we can design lower-order methods with convergence rates overpassing the classical complexity bounds for convex composite problems. Hence, this will open up an entirely new ground for developing novel efficient algorithms for convex composite optimization that was not possible in the classical complexity theory.

Several extensions of our framework are possible. As an example, we will present some extension of our framework using a segment search in the upcoming article [3]. Moreover, the proximal-point auxiliary problem can be solved by some more efficient method such as the non-Euclidean line search method [5] with second-order directions [4, 6, 34]. In addition, the introduced high-order scaling function can be employed to extend the second-order methods presented in [27–30] to higher-order methods.
